# Integrating Exposure and Effect Distributions with the Ecotoxicity Risk Calculator: Case Studies with Crop Protection Products

**DOI:** 10.1002/ieam.4344

**Published:** 2020-10-30

**Authors:** David A Dreier, Sara I Rodney, Dwayne RJ Moore, Shanique L Grant, Wenlin Chen, Theodore W Valenti, Richard A Brain

**Affiliations:** ^1^ Syngenta Crop Protection, LLC Greensboro North Carolina USA; ^2^ Intrinsik Ottawa Ontario Canada; ^3^ Intrinsik New Gloucester Maine USA

**Keywords:** Probabilistic approaches, Species sensitivity distributions, Ecological risk assessment, Risk curves, Joint probability curves

## Abstract

Risk curves describe the relationship between cumulative probability and magnitude of effect and thus express far more information than risk quotients. However, their adoption has remained limited in ecological risk assessment. Therefore, we developed the Ecotoxicity Risk Calculator (ERC) to simplify the derivation of risk curves, which can be used to inform risk management decisions. Case studies are presented with crop protection products, highlighting the utility of the ERC at incorporating various data sources, including surface water modeling estimates, monitoring observations, and species sensitivity distributions. *Integr Environ Assess Manag* 2021;17:321–330. © 2020 Syngenta Crop Protection, LLC. *Integrated Environmental Assessment and Management* published by Wiley Periodicals LLC on behalf of Society of Environmental Toxicology & Chemistry (SETAC)

## KEY POINTS

- Risk curves describe the relationship between distributions of exposure and effect.
- The Ecotoxicity Risk Calculator can be used to generate risk curves from various data sources.
- Case studies are presented with risk curves for crop protection products.

## INTRODUCTION

Probabilistic approaches are often used in higher tiers of ecological risk assessment. For example, species sensitivity distributions (SSDs) are widely used for effects characterization to infer hazardous concentrations (HCs) predicted to result in the exceedance of toxicity endpoints (e.g., LC50s or NOECs) for a specified percentage of species in a community (Newman et al. 2000). Applications of SSDs have been diverse (Posthuma et al. 2001), including seminal examples for agrochemicals by Klaine et al. (1996) and Solomon et al. (2000). Such techniques have also been adapted for other various industrial compounds, personal care products (Capdevielle et al. 2008), and pharmaceuticals (Zhao et al. 2017), as well as nonchemical stressors such as temperature (de Vries et al. 2008) and hypoxia (Saari et al. 2018). Additionally, distributions of exposure have been derived from several tiers of prospective surface water modeling, as well as monitoring datasets (Travis and Hendley 2001; Mosquin et al. 2012). Several tiers of surface water modeling exist, and refined models can include landscape, physical‐chemical, and fate properties, among others. In certain situations, more realistic estimates through monitoring data are required, as sophisticated models used in the context of chemical safety assessments typically overestimate exposure (ECOFRAM 1999). Predicted and observed concentrations usually vary spatially as well as temporally, yielding distributions of exposure data. Such distributions offer a comprehensive synthesis of exposure, and the selection of specific data sets (i.e., those varying spatially, temporally, or both) is dictated by the circumstances of the risk assessment. For example, exposure distributions varying spatially can inform risk assessments with geographic boundaries (i.e., those for threatened and endangered species), whereas exposure distributions varying temporally are valuable to ascertain the frequency of acute effects or likelihood of chronic effects. Distributions encompassing data varying both spatially and temporally can also be used in regional or national assessments and for global scanning exercises to identify common exposure patterns and outliers.

The integration of effect and exposure distributions offers a powerful approach to assess, communicate, and manage risks. A common format is the risk curve, also termed a joint probability curve, which has been used primarily for data‐rich substances such as agrochemicals (Giesy et al. 1999; Giddings et al. 2000; Moore et al. 2010) and certain industrial compounds (Moore et al. 2016). Given the availability of both effect and exposure distributions, risk curves depict the relationship between levels of predicted effects and the probability of exceeding them (Solomon et al. 2000). Risk curves contain far more information than do deterministic risk quotients, defined by point estimates of exposure and effect data, and hence are more useful for informing higher‐tier risk assessment and/or risk management decisions. Contrary to quotient‐based methods, risk curves can be used to investigate how various management strategies may reduce risk given that both exposure and effect data are distributed, such that risk is also distributed in space and/or time. With these considerations, risk curves are recommended in higher‐tier ecological risk assessments (ECOFRAM 1999), including those for threatened and endangered species in which many sources of variability and uncertainty are anticipated (NRC 2013; Brain et al. 2015).

Although risk curves offer a robust approach, their adoption has remained limited in practice, because while conceptually simple, development of a joint probability is considered complex. In many cases, specialized software packages are required to derive risk curves, thereby limiting their application. There has also been a paucity of guidance informing what constitutes acceptable risk in the context of probability by regulators, a necessary prerequisite for adopting probabilistic risk assessment tools. To improve the accessibility and adoption of this approach, a publicly available Ecotoxicity Risk Calculator (ERC) was developed to facilitate the construction and interpretation of risk curves. The purpose of this manuscript is to highlight the utility of the ERC using a selection of case studies, and to make the tool openly accessible to risk assessors, managers, and the greater scientific community.

## METHODS

### Data compilation for case studies

Crop protection products are among the most intensively studied compounds and many have a wealth of effects and exposure data available that are “fit for purpose” and supportive for advancing to higher tiers of risk characterization. As such, several case studies were developed with crop protection products to illustrate the utility of the ERC. Active ingredients were selected from different use classifications to include an insecticide (thiamethoxam), fungicide (chlorothalonil), and herbicide (atrazine). Published data sets were compiled where available, and data were removed that would not commonly be used in current regulatory assessments (i.e., those not meeting quality assurance or control criteria). The purpose of this work is to highlight the potential application of the ERC tool and hence select case studies focused on compounds from which an extensive amount of prior work had been completed. A summary of the data sets used in each case study is provided in Table 1, and further details are presented in following sections.

**Table 1:** Data sources used to assemble risk curves for each case study

Active ingredient	Use classification	Risk component	Data source	Reference(s)
Thiamethoxam	Insecticide	Effects	Species sensitivity distribution for acute aquatic invertebrate toxicity	Finnegan et al. 2017; PMRA 2018
		Exposure	US Environmental Protection Agency (USEPA) Surface Water Concentration Calculator	Inputs from Bartell et al. 2018
		Exposure	Monitoring observations	Finnegan et al. 2017
Chlorothalonil	Fungicide	Effects	Species sensitivity distribution for acute fish toxicity	Brain et al. 2010
		Exposure	USEPA Pesticide in Water Calculator	NA
		Exposure	Water quality portal	NA
Atrazine	Herbicide	Effects	Species sensitivity distribution for acute aquatic plant toxicity	Erickson 2012
		Exposure	USEPA Pesticide in Water Calculator	Inputs from Giddings et al. 2005
		Exposure	Atrazine Ecological Exposure Monitoring Program	USEPA 2019a

Case Study #1 involved the neonicotinoid insecticide thiamethoxam, for which modeling and monitoring exposure distributions were available from previous studies. Briefly, the US Environmental Protection Agency (USEPA) Surface Water Concentration Calculator (now called the Pesticide in Water Calculator [PWC]) was used to generate the modeling exposure distribution representing standard regions and crop scenarios for thiamethoxam use in the USA (Bartell et al. 2018). Thiamethoxam surface water concentrations were computed in a 30‐y simulation for a standard water body (EPA farm pond, 1‐ha area by 2‐m depth), and daily peak values from the California citrus scenario (with 0.096 kg a.i./ha, 2 applications, and 7‐d application interval) were used to define the modeling exposure distribution in the ERC. The monitoring exposure distribution was originally described by Finnegan et al. (2017) and comprises numerous data sets, including those from the peer‐reviewed literature, published reports, and online databases. Observations from various water body types were included in the distribution, including streams, ponds, lakes, and wetlands. Aquatic invertebrates were selected for the SSD because they are the most sensitive tested taxa to neonicotinoid insecticides, such as thiamethoxam, and therefore provide information for a relatively sensitive taxonomic group. The SSD was derived from acute toxicity values published in a special review of thiamethoxam by the Pest Management Regulatory Agency (PRMA) in Canada, which includes the most comprehensive SSD for aquatic invertebrates published to date (PMRA 2018) (Supplemental Data Table S1).

Case Study #2 employed a data mining approach to derive a monitoring exposure distribution for the fungicide chlorothalonil. Briefly, water quality data including chlorothalonil concentrations were downloaded on 2 October 2019 from the water quality portal managed by the National Water Quality Monitoring Council (Read et al. 2017). Chlorothalonil was selected as a sampling parameter under “Characteristics,” and further data processing was conducted with the downloaded CSV file for “Sample results (physical/chemical metadata)”. A custom R script was run to select routine surface water samples, remove duplicates, and adjust concentration units. To promote consistency among analytical approaches, recent observations between 1 January 2008 and 31 December 2018 were retained for further analysis. When more than 1 observation was reported within a 24‐h period at a sampling location, the maximum value was retained to acknowledge that higher exposures may induce effects that potentially remain after the exposure event, thus better reflecting the likelihood of potential impacts in the risk curve. Observations were only retained if the limit of detection and/or quantification were reported. Undetectable concentrations were assigned a value equal to one‐half of the detection quantitation limit measure reported for the sample. Samples were considered regardless of geographic location to develop an ambient exposure distribution. To generate the modeling exposure distribution, the CA potatoes scenario was implemented in PWC with an application rate of 1.26 kg a.i./ha, 10 applications, and 5‐d application interval. Finally, an SSD for fish was generated based on a previous assessment for endangered and threatened species (Brain et al. 2010) (Supplemental Data Table S2). To reduce variability as a function of exposure duration, the SSD only included 96‐hour observations, as this duration comprised the majority of acute toxicity observations. The geometric mean was used if more than 1 study was available per species. As before, this SSD provides a conservative estimation of hazard, as fish have notable sensitivity to chlorothalonil.

Case Study #3 compared modeling and monitoring exposure data for the herbicide, atrazine. Environmental fate parameters reported by Giddings et al. (2005) were used to parameterize the PWC model to generate a daily exposure profile for the Nebraska Corn scenario. Two (2) ground applications (at 2.24 kg a.i./ha and 0.56 kg a.i./ha) were assumed with a 28‐d application interval. Monitoring data were compiled from the Atrazine Ecological Exposure Monitoring Program (Chen et al. 2016; USEPA 2019a). Recent observations (2014–2018) were selected from the monitoring database for 9 headwater watersheds targeting intensive atrazine use with high runoff vulnerability for corn production in Iowa, Texas, Missouri, Louisiana, and Nebraska. Like thiamethoxam and chlorothalonil, the maximum value was retained for multiple observations within a 24‐h period at a monitoring location. Following compilation, monitoring exposure distributions were integrated with an SSD for acute toxicity in aquatic plants (Erickson 2012; Moore et al. 2020) (Supplemental Data Table S3). Aquatic plants are sensitive to herbicides such as atrazine, and therefore the risk curve is expected to be protective of consumer species (e.g., fish and invertebrates).

### Ecotoxicity risk calculator

**Figure 1:**
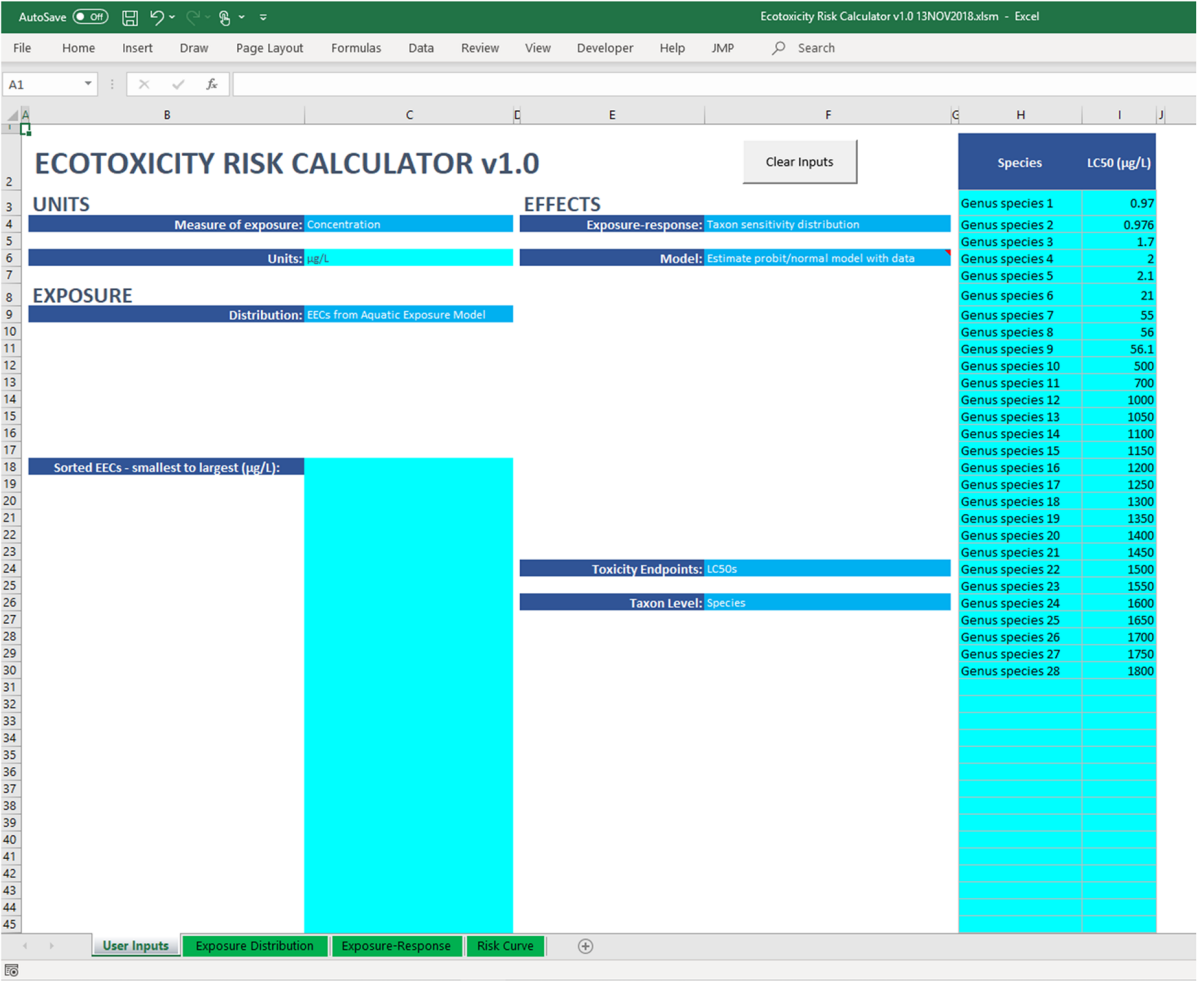
“User Inputs” data entry worksheet for estimating a taxon sensitivity distribution.

The ERC was used to derive a risk curve by integrating exposure and effect distributions for each case study. The ERC is implemented in Microsoft Excel (Figure 1) to increase accessibility for risk assessors; the tool and user manual are provided in the Supplemental Data for wider use. The program can be used to define exposure and effect distributions using various approaches. For example, a list of estimated exposure concentrations (EECs) can be added to define the exposure distribution from modeling (e.g., PWC output) or monitoring data, or the distribution can be estimated from various 2‐parameter models (e.g., lognormal, normal, Weibull). In addition, effects data can be added for a single species to define an exposure‐response distribution considering a particular endpoint (e.g., survival, growth, reproduction), or a taxon sensitivity distribution can be added. The effects distribution can be derived from raw data or estimated using various models (e.g., Gompertz, Gumbel, logistic, probit, Weibull). For each case study, the exposure distribution was defined using a list of values compiled from either modeling or monitoring studies. Similarly, an SSD was fit with a probit model in the ERC to estimate the effects distribution. A modified Anderson‐Darling statistic was implemented to determine goodness‐of‐fit, and *p*‐values ≥0.1 were considered acceptable. Finally, water quality criteria were added to each figure to provide additional context. Aquatic life benchmarks from the USEPA (USEPA 2020) were added for thiamethoxam (acute aquatic invertebrate toxicity = 17.5 μg/L) and chlorothalonil (acute fish toxicity = 5.25 μg/L), whereas the concentration equivalent level of concern was added for atrazine (aquatic plant toxicity = 15 μg/L) (USEPA 2019b). These aquatic life criteria are intended to be conservative estimates of concentrations that are protective of aquatic biota. The purpose of citing these criteria here is for comparison or reference only.

A risk curve was generated by calculating the percent effect and probability of exceedance for a series of concentrations. For each concentration, these values (percent effect and probability of exceedance) are then plotted against each other to generate the risk curve. Summary statistics for the risk curve include area under the curve (AUC) and maximum risk product (MRP). The AUC is defined as the integral of the risk curve from 0 to 100% effect, whereas the MRP is the highest product (expressed as a percent) of probability of exceedance and magnitude of effect from the risk curve. To provide context, lines of equivalent risk product were drawn based on Moore et al. (2010) to define boundaries for de Minimis‐Low risk (MRP = 0.25%; AUC = 1.75%), Low‐Intermediate risk (MRP = 2%; AUC = 9.82%), and Intermediate‐High risk (MRP = 10%; AUC = 33.0%).

## RESULTS

**Figure 2:**
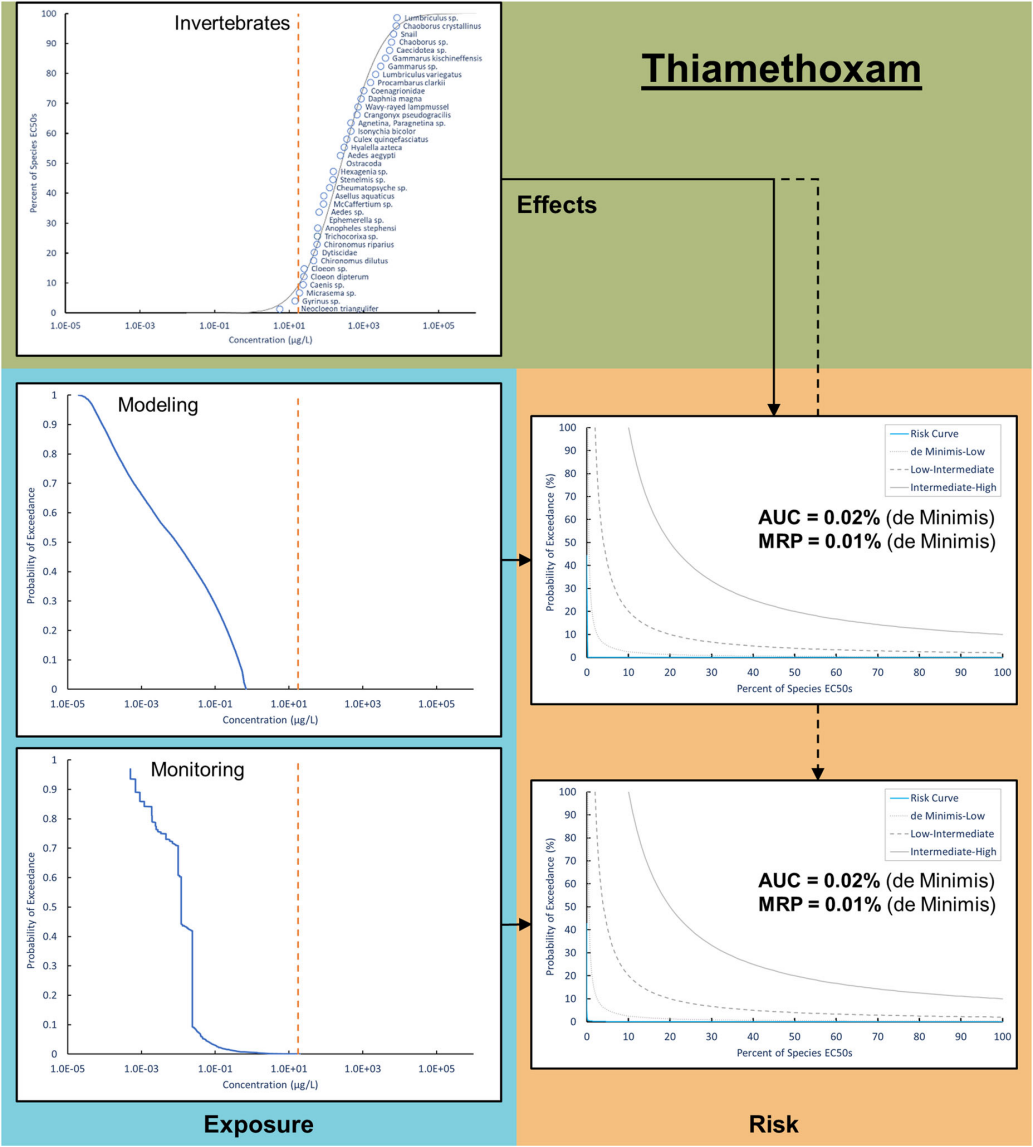
A species sensitivity distribution for aquatic invertebrates integrated with modeling and monitoring exposure distributions to estimate risk curves for thiamethoxam. The dashed orange line represents the US Environmental Protection Agency (USEPA) aquatic life benchmark (point estimate) for thiamethoxam acute toxicity in aquatic invertebrates (17.5 μg/L).

The first case study with thiamethoxam included a comprehensive acute SSD for aquatic invertebrates (Figure 2). In total, 37 species were considered, and the HC5 of the distribution (i.e., the concentration representing the 5th percentile of toxicity values, equivalent to a 95% protection level) was 9.21 µg/L. The goodness‐of‐fit was acceptable, with an adjusted Anderson‐Darling statistic of 0.53 and *p*‐value of 0.18. The most sensitive species was *Neocloeon triangulifer* with an EC50 of 5.5 µg/L, whereas the least sensitive organism was *Lumbriculus sp*., which had an EC50 of 7700 µg/L. The modeling exposure distribution included 10 951 nonzero values ranging from 2.00 × 10^−5^ to 1.44 µg/L, and the monitoring dataset included 6906 observations, ranging from 5.00 × 10^−4^ to 225 µg/L. When the effect and modeling exposure distributions were integrated, the AUC = 0.02% and MRP = 0.01%, representing de minimis risk for acute toxicity in aquatic invertebrates. The risk curve integrating monitoring exposure values with the SSD had almost identical estimates for the AUC and MRP.

**Figure 3:**
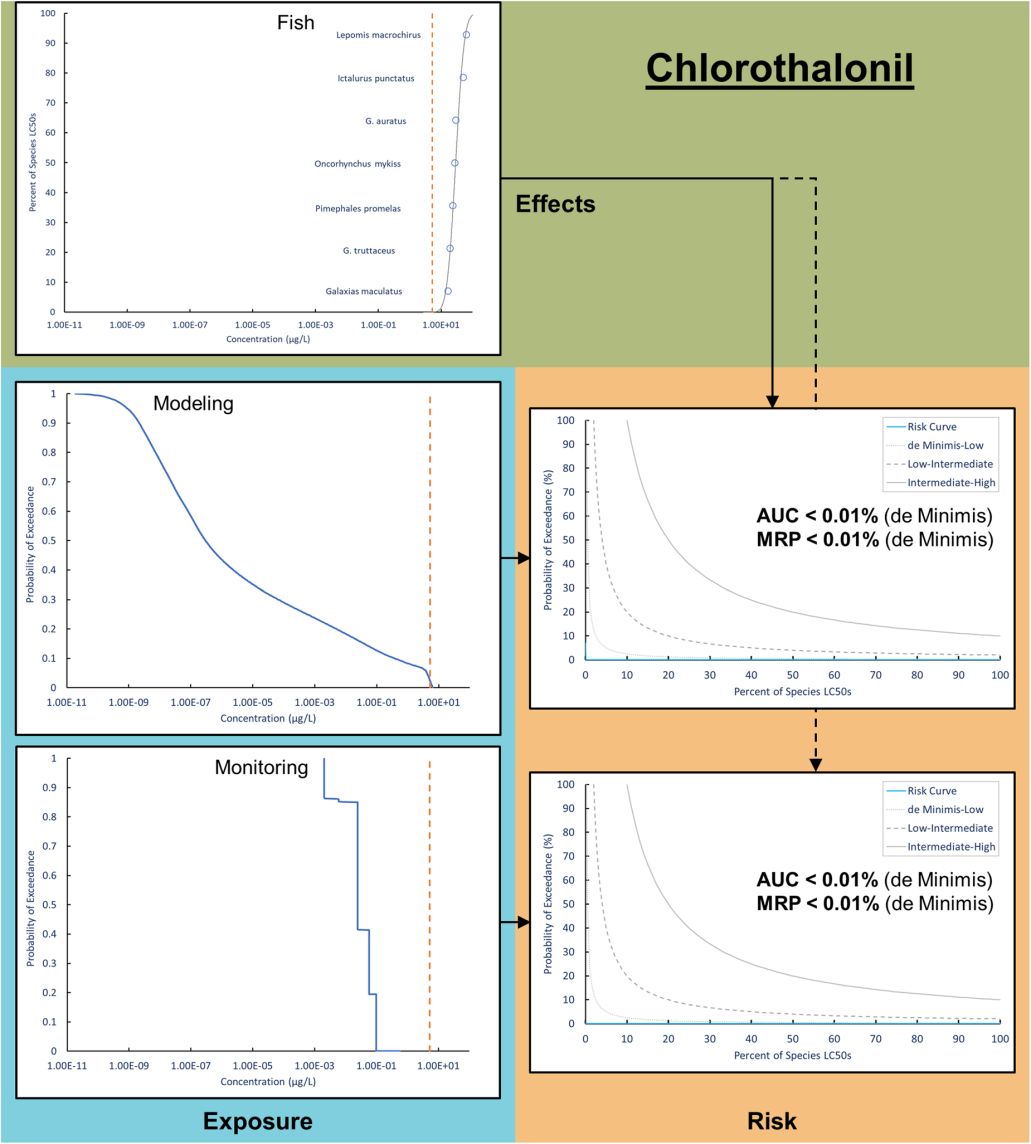
A species sensitivity distribution for acute fish toxicity integrated with modeling and monitoring exposure distributions to estimate risk curves for chlorothalonil. The dashed orange line represents the US Environmental Protection Agency (USEPA) aquatic life benchmark (point estimate) for chlorothalonil acute toxicity in fish (5.25 μg/L).

The second case study with chlorothalonil used an acute toxicity SSD for fish (Figure 3). The SSD included 7 fish species, and the HC5 of 12.8 µg/L was lower than the EC50 of the most sensitive species, *Galaxias maculatus*, at 16.3 µg/L. The goodness‐of‐fit was acceptable, with an adjusted Anderson‐Darling statistic of 0.32 and *p*‐value of 0.44. The modeling exposure distribution included 10 853 nonzero values ranging from 1.91 × 10^−11^ to 16.2 µg/L. The monitoring exposure distribution primarily consisted of nondetect values and ranged from 2.00 × 10^−4^ to 3.09 µg/L. In total, there were 877 monitoring locations and 7216 unique samples, which occurred primarily in the spring and summer concordant with the growing season. When integrated with effects data, the modeling and monitoring exposure distributions led to risk curves with an AUC and MRP < 0.01%, representing de minimus risk for acute toxicity in fish.

**Figure 4:**
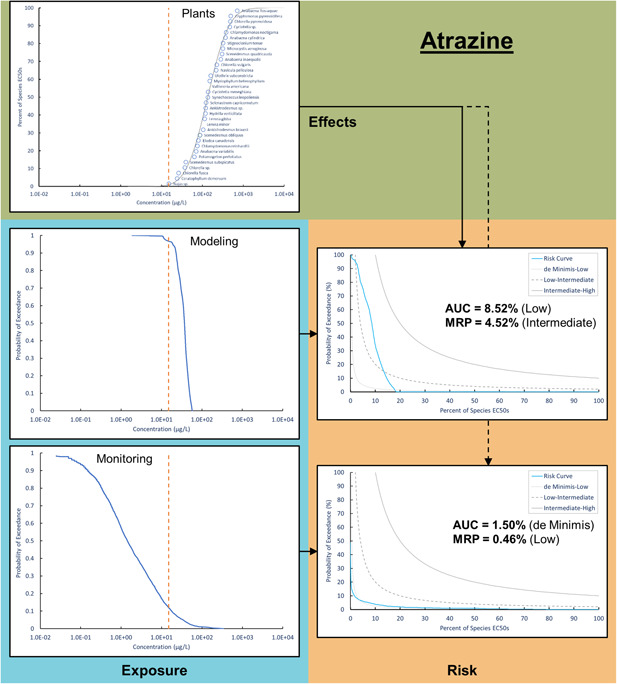
A species sensitivity distribution for aquatic plants integrated with modeling and monitoring exposure distributions to estimate risk curves for atrazine. The dashed orange line represents the US Environmental Protection Agency (USEPA) concentration equivalent level of concern (point estimate) for atrazine toxicity in plants (15 μg/L).

The third case study employed an aquatic plant SSD for atrazine (Figure 4). The SSD included 33 species, with EC50 values ranging from 15 to 706 µg/L and resulting HC5 of 28.5 µg/L. The goodness‐of‐fit was acceptable, with an adjusted Anderson‐Darling statistic of 0.33 and *p*‐value of 0.41. The PWC modeling exposure distribution included 10 827 nonzero values ranging from 1.86 to 86.6 µg/L, whereas the monitoring exposure distribution included 4691 values ranging from 0.025 to 344 µg/L. When modeling data were used to estimate exposure, the risk curve AUC was 8.52% (low risk) and MRP was 4.52% (intermediate risk). However, when monitoring data were employed, the AUC and MRP declined to 1.50% and 0.46%, representing de minimis and low risk, respectively, for aquatic plant toxicity.

## DISCUSSION

Probabilistic approaches are an important component of higher‐tier ecological risk assessment. Although not new, the adoption of risk curves has remained limited in practice compared to deterministic methods, such as risk quotients, for which specialized software tools are not required. In this manuscript, we highlight the utility and convenience of the ERC tool in Microsoft Excel with a selection of case studies involving crop protection products.

The case studies provided herein examined agrochemicals for several use classifications, as robust, transparent, and accessible data sets were available to support probabilistic approaches. For each compound, an SSD was constructed for the most sensitive taxonomic group based on mode of action, from which a conservative estimate of hazard could be generated (e.g., the HC5). Modeling and monitoring exposure distributions were also compiled for each compound to facilitate comparisons. Notably, each of the modeling exposure distributions spanned several orders of magnitude, including concentrations at which monitoring detections were also recorded. However, most exposure monitoring observations were undetectable, especially for thiamethoxam and chlorothalonil, and were therefore assigned a conservative value of one‐half of the limit of detection or quantification. This approach was employed instead of removing undetectable concentrations, because it offers a more realistic distribution of exposure by including observations with low concentrations (Solomon et al. 2000). Accordingly, many exposure modeling estimates are below these limits of detection or quantification. In contrast, each of the models typically overestimated the probability of exceedance at higher concentrations, highlighting the conservative nature of such approaches (Levine et al. 2019).

The risk curve generated from surface water models for atrazine indicated intermediate risk, whereas the use of higher‐tier monitoring data indicated low risk. The mismatch in the risk characterization underlines the dislocation of the 2 exposure distributions generated by the model predictions and the actual measurement data. The Nebraska Corn scenario was selected in PWC to offer a regulatory assessment model for atrazine under a set of hypothetical worst‐case conditions including maximum label use rate on the same day application over entire watersheds for 30 y. The higher‐tier monitoring data in this case provide a synthesis of intensive monitoring efforts focused on watersheds representing the upper 20^th^ percentile of runoff vulnerability as designed by the Atrazine Ecological Monitoring Program. Almost the entire exposure distribution predicted by the PWC model is narrowly located on the upper tenth percentile of the measured distribution (Figure 4), suggesting severe overprediction by the PWC model. Clearly, there are opportunities to refine hazard characterization through higher‐tier modeling approaches informed by monitoring data. Compared to point estimates (e.g., NOEC/LOEC) for the most sensitive species, the inclusion of multiple species in an SSD offers a more comprehensive approach to characterize hazard for a taxon of interest. For example, *Najas sp*. was most sensitive among tested species to atrazine with an EC50 of 15 µg/L, yet most aquatic plants in the SSD had an EC50 >100 µg/L, with a maximum EC50 of 706 µg/L. Considering that functional redundancy may exist among these organisms in an ecosystem (Walker 1992; Baskin 1994; Moore 1998), SSDs offer a refined approach to estimate aquatic hazard, especially when ecological risk assessments define protection goals at higher levels of biological organization.

The ERC is a useful tool to describe the risk curves of various use scenarios to identify the scenario representing the lowest likelihood of potential impacts. For example, multiple PWC scenarios can be modeled for a given compound, and the resulting AUC and MRP from the risk curves compared. Such an approach could be used to identify the scenario representing the lowest potential impact, thereby supporting good agricultural practices by region and use. Although the case studies presented in this manuscript employed SSDs to characterize hazard, the ERC can use single species exposure‐response relationships. For example, risk curves have been used to determine the probability of brain acetylcholinesterase inhibition in juvenile salmon given a defined exposure distribution of dimethoate (Whitfield‐Aslund et al. 2017) and other carbamates and organophosphates (Moore and Teed 2013). Assessments such as these can characterize the probabilities of certain effects, which could then be linked to existing modeling approaches that support ecological risk assessment, such as quantitative adverse outcome pathways (Perkins et al. 2019), dynamic energy budget models (Murphy et al. 2018), and population models (Forbes et al. 2008).

In addition to these applications in risk assessment, the ERC also has utility to inform risk management decisions. Compared to deterministic approaches (e.g., quotient method), risk curves can be used to predict both the magnitude and likelihood of an effect. With this information, one can evaluate the effectiveness of various risk management options by determining the degree of risk reduction from changes in exposure. However, it remains challenging to identify appropriate management trigger values for risk curves, especially relative to protection goals established by regulatory authorities. Joint probability curves incorporate a variety of sources of uncertainty in a risk assessment (e.g., from sampling, biological variability, measurement error). Following a probabilistic approach, the variability and/or incertitude of inputs can be propagated through exposure and effect distributions and subsequently integrated in a risk curve (NRC 2013). Because the ERC can easily integrate such data (e.g., AUC, MRP), it also provides an important tool to communicate uncertainty to risk managers.

## CONCLUSIONS

The ERC provides an openly accessible tool to derive risk curves based on defined exposure and effect distributions. As a probabilistic approach, risk curves offer a full description of the relationship between cumulative probability and magnitude of effect, thus greater utility than deterministic approaches for risk assessment and management. Moving forward, we expect the ERC will lead to increased adoption of risk curves in ecological risk assessments for agrochemicals, industrial compounds, and pharmaceuticals, as well as other potential environmental stressors. This adoption will be necessary to support ecological risk assessments with specific challenges and/or numerous sources of uncertainty, such as those for threatened or endangered species. We recommend that risk curves become the common format to communicate ecological risks, thereby improving management decisions.

## Disclaimer

The peer‐review process for this article was managed by the editorial board without the involvement of Dwayne RJ Moore and Richard A Brain.

## SUPPLEMENTAL DATA

In the Supplemental Data, readers can find supplemental tables as well as files containing the following:

Ecotoxicity Risk Calculator v1.0 13NOV2018.xlsx: Excel‐based risk curve tool.

Ecotoxicity Risk Calculator v1.0—User's Manual 13NOV2018.pdf: User manual for Excel‐based risk curve tool.

Atrazine—Modeling.xlsm: Atrazine case study data (modeling exposure distribution).

Atrazine—Monitoring.xlsm: Atrazine case study data (monitoring exposure distribution).

Chlorothalonil.xlsm—Modeling: Chlorothalonil case study data (modeling exposure distribution).

Chlorothalonil—Monitoring.xlsm: Chlorothalonil case study data (monitoring exposure distribution).

Thiamethoxam—Modeling.xlsm: Thiamethoxam case study data (modeling exposure distribution).

Thiamethoxam—Monitoring.xlsm: Thiamethoxam case study data (monitoring exposure distribution).

## Supporting information

This article contains online‐only Supplemental Data.

Supporting information.

Supporting information.

Supporting information.

Supporting information.

Supporting information.

Supporting information.

Supporting information.

Supporting information.

Supporting information.

## Data Availability

Data and associated metadata and calculation tools are available upon request by contacting corresponding author David A Dreier (david.dreier@syngenta.com).
