# Supplementary material for: Integrating Exposure and Effect Distributions with the Ecotoxicity Risk Calculator: Case Studies with Crop Protection Products
Source: Integr Environ Assess Manag. 2020 Oct 30;17(2):321–30. doi: 10.1002/ieam.4344 (PMC7894161; doi:10.1002/ieam.4344)
Supplement: Supplementary file 5 — Supporting information. [file IEAM-17-321-s005.pdf]

TABLE OF CONTENTS

1.0 Introduction ... 5
2.0 Getting Started ... 7
3.0 Selecting Appropriate Units ... 8
4.0 Entering an Exposure Distribution ... 8
5.0 Entering Effects Information ... 11
4.1. Option 1: Parametric Exposure-Response Distributions ... 12
4.2. Option 2: Binomial Effects Data ... 15
4.3. Option 3: Taxon-Specific Toxicity Endpoints ... 17
6.0 Risk Curve ... 19
7.0 Computational Details ... 21
7.1. Exposure Distributions ... 21
7.2. Exposure-Response Distributions Based on User Input Parameters ... 22
7.3. Probit Analysis for Binary Effects Data ... 23
7.4. Taxon Sensitivity Distribution Based on Entered Toxicity Endpoints ... 25
7.5. Risk Curve Calculations ... 27
8.0 References ... 29

1.0 INTRODUCTION

Probabilistic ecological risk assessment typically involves estimating distributions of potential exposure concentrations or doses for receptors of concern. Such distributions may be combined with exposure-response distributions to derive risk curves. Exposure-response curves are generally derived from toxicity data for a representative laboratory test species. For communities, species or taxon sensitivity distributions may be used.

Risk curves or joint-probability curves have been recommended and used in recent refined risk assessments (e.g., Moore et al., 2010, 2014, 2015; NRC, 2013; Brain et al., 2015; Clemow et al. 2018). A risk curve indicates the probabilities of exceeding levels of effect of differing magnitude (Figure 1). Figure 1, for example, indicates that there is an 25% probability of exceeding 5% mortality, and only a 5% probability of exceeding 10% mortality.

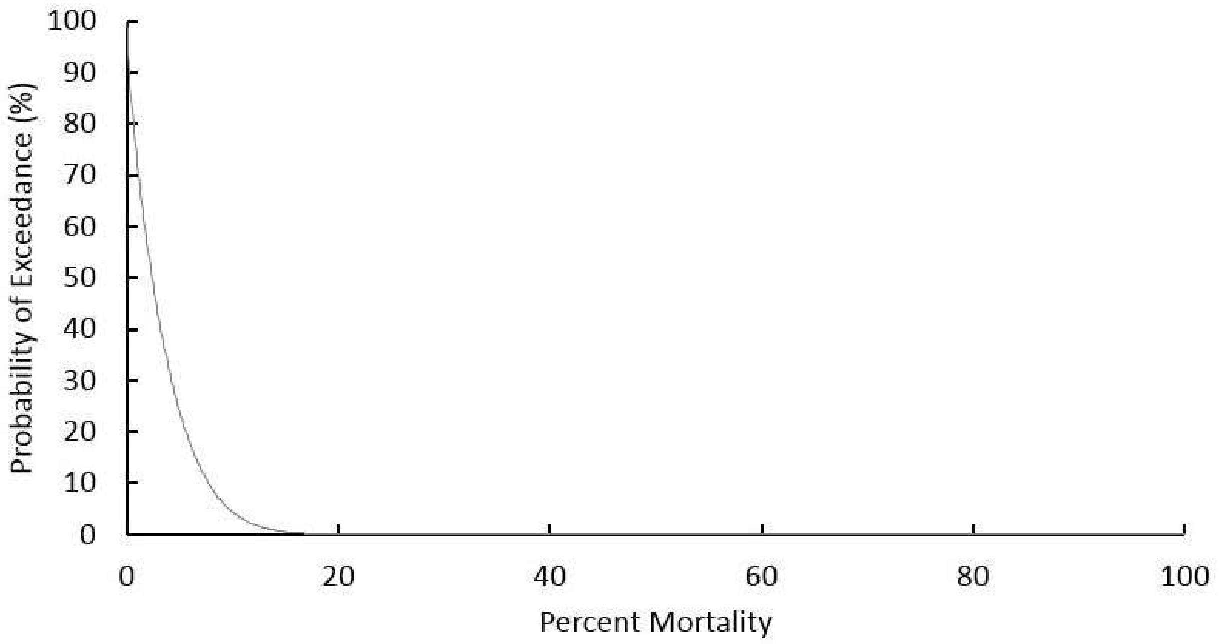

Figure 1. Example of a risk curve or joint probability curve.

An impediment to widespread quantitative probabilistic risk characterization is the lack of easily accessible and user-friendly tools for estimating and integrating exposure and effects distributions. MS Excel® is a ubiquitous spreadsheet-based application available to all or nearly all risk assessors. In MS Excel®, risk curves can be readily generated from specified exposure and exposure-response and distributions.

The Ecotoxicity Risk Calculator (Ecotoxicity Risk Calculator) v1.0 described herein is a basic tool for generating risk curves for probabilistic ecological risk assessment. The tool is expandable and additional capabilities can be added over time to meet the needs of users. The current Ecotoxicity Risk Calculator v1.0 features include:

1) Exposure: From a list of available two-parameter distributions, select and parameterize an appropriate distribution, or input a set of estimated exposure concentrations (EECs; e.g., from PRZM/EXAMS or other exposure model).
2) Effects:
    a) From a list of available two-parameter distributions, select and parameterize an appropriate distribution, or
    b) Input raw data and select the type of effects data (e.g., binomial or taxon toxicity endpoints).
        i) For a dataset of toxicity endpoints (e.g., EC50s for a species sensitivity distribution), a normal univariate MLE approach is used to determine parameters. The Anderson-Darling statistic is calculated to assess goodness-of-fit.
        ii) For binomial responses (e.g., mortality, presence/absence), a Maximum Likelihood Estimation (MLE) approach assuming a probit distribution is used. The Pearson-Chi-square statistic is calculated to assess goodness-of-fit.
3) A risk curve is generated based on the user-selected exposure and effects distributions and the results are presented in table and figure formats. Summary statistics including area under the curve (AUC) and maximum risk product are given. The AUC is the integral of the risk curve from 0 to 100% effect. The maximum risk product is the highest product (expressed as a percent) of probability of exceedance and magnitude of effect from the risk curve.

Sections 2 to 6 describe how to use the Ecotoxicity Risk Calculator v1.0. Section 7 provides the computational details employed in the calculator, including the equations that define the exposure distributions and exposure-response distributions.

2.0 GETTING STARTED

The Ecotoxicity Risk Calculator v1.0 is a macro-enabled Excel workbook. For the workbook to operate properly:

1) Macros must be enabled (Macro Security→Macro Settings→Enable all macros)
2) The Solver add-in must be enabled (File→Options→Add-ins. Select Solver Add-in)
3) In Visual Basic, you must include reference to Solver (Developer→Visual Basic→Tools→References. Check the Solver reference.)

The same Ecotoxicity Risk Calculator v1.0 excel file can be used repeatedly by simply deleting the input data on the worksheet titled "User Inputs" and starting over again. This can be done by pressing the "Clear Inputs" button on the "User Inputs" worksheet (See Figure 1).

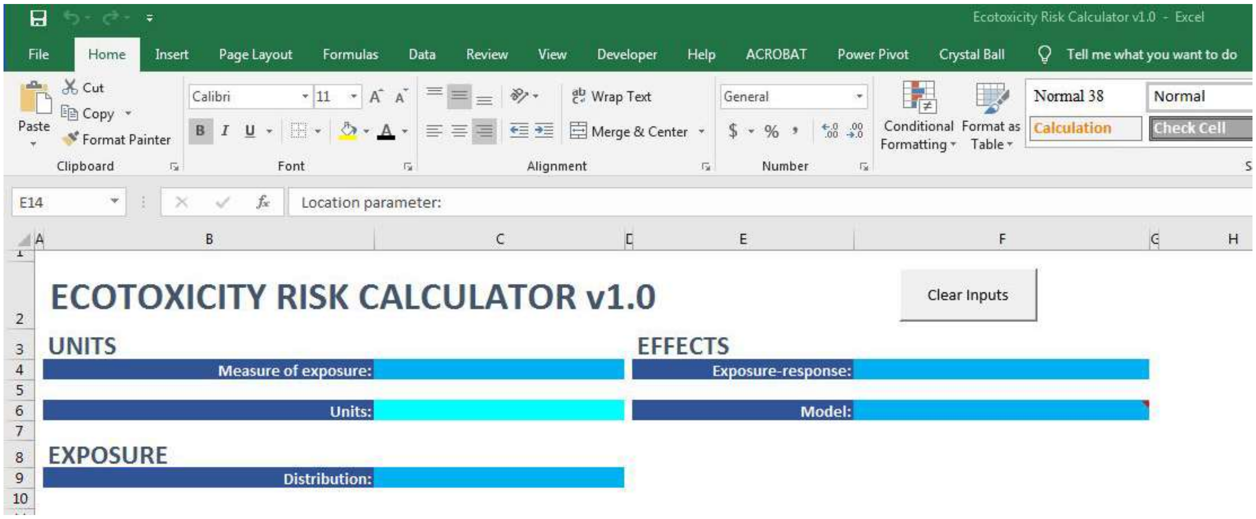

Figure 1. "User Inputs" worksheet showing initial input fields and the "Clear Inputs" button which clears all information entered by the user, so that the file can be updated.

To generate a risk curve, exposure and exposure-response distributions are required. In the Ecotoxicity Risk Calculator v1.0, the user can enter distribution parameters for one of three exposure distribution distributions (normal, lognormal or Weibull). Alternatively, the user can enter a set of estimated exposure concentrations (EECs), such as PRZM/EXAMS output.

For the exposure-response distribution, the user can enter distribution parameters for a suite of possible exposure-response distributions (i.e., probit/normal, logistic, Gompertz/extreme value, Gumbel, Weibull). In all cases, except the Weibull distribution (which is constrained to positive values), distributions are based on log10(exposure), to avoid predicting effects when exposure is zero or less and also because distribution fit is generally much better in log space. Equations for exposure distributions and exposure-response distributions are given in Section 7 (Computational Details).

The Ecotoxicity Risk Calculator v1.0 can also fit distributions to empirical binomial single species data (e.g., mortality) or taxon sensitivity distributions (e.g., dataset of LC50s for different species). Thus, these data can be used instead of a pre-defined exposure-response distribution.

To start, open the workbook and ensure that the input fields are clear by pressing the "Clear Inputs" button (Figure 1).

3.0 SELECTING APPROPRIATE UNITS

The user must select the type of exposure being considered in cell C4 on the "User Inputs" worksheet (e.g., concentration, body burden, daily intake rate, application rate, etc.). Both exposure and exposure-response must be the same units to enable proper generation and interpretation of the risk curve, and thus there is only one cell in which to enter units (C6 on the "User Inputs" worksheet; Figure 2).

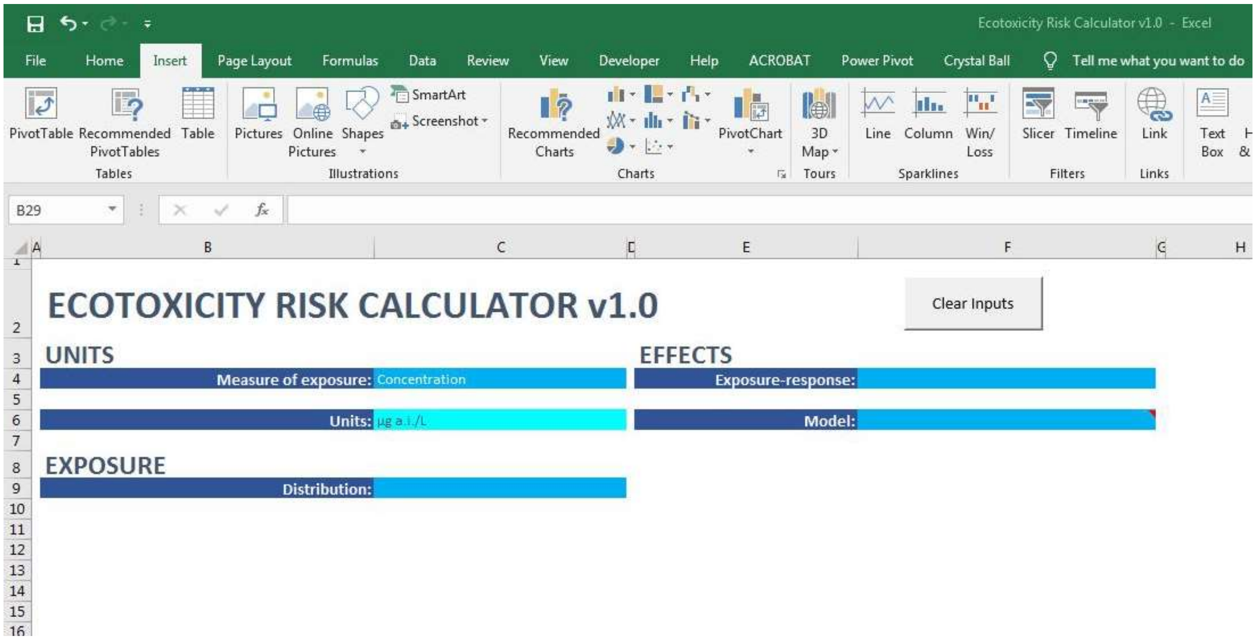

Figure 2. "User Inputs" worksheet showing for example, the selection of "Concentration" as the measure of exposure and specified units of µg a.i./L.

4.0 ENTERING AN EXPOSURE DISTRIBUTION

The next step in generating a risk curve is to input an exposure distribution. The user can either enter a set of exposure values (e.g., output from PRZM/EXAMS) or select an appropriate parametric exposure distribution (cell C9 on the "User Inputs" worksheet).

To enter a set of exposure values, select "Set of EECs" as the distribution from the drop-down list in cell C9. A space will appear in the column below where the exposure values can be entered. In column C, enter the exposure values from smallest to largest starting at cell C18 (in the range of cyan cells; Figure 3).

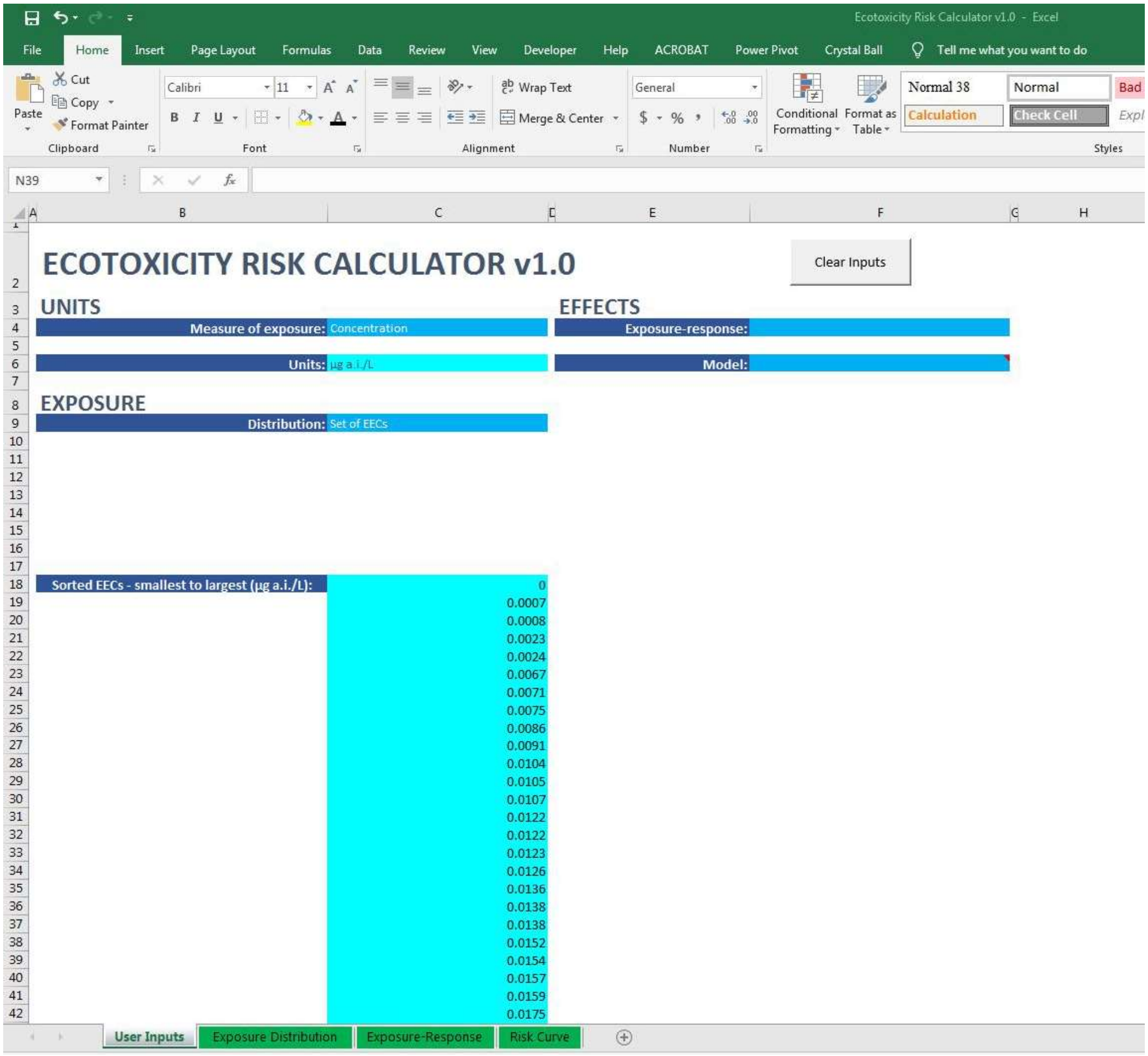

Figure 3. **"User Inputs" worksheet showing, for example, the input of a set of EECs as the exposure distribution.**

Alternatively, the user may define a parametric distribution. This is a function that describes exposure data based on distribution model-specific parameters. The drop-down list in cell C9 includes possible model distributions. The user must select one from the list and then enter the necessary distribution parameters. The user may select from the normal, lognormal and Weibull distributions (Figure 4). The parametric distributions are described in more detail in Section 7 (Computational Details).

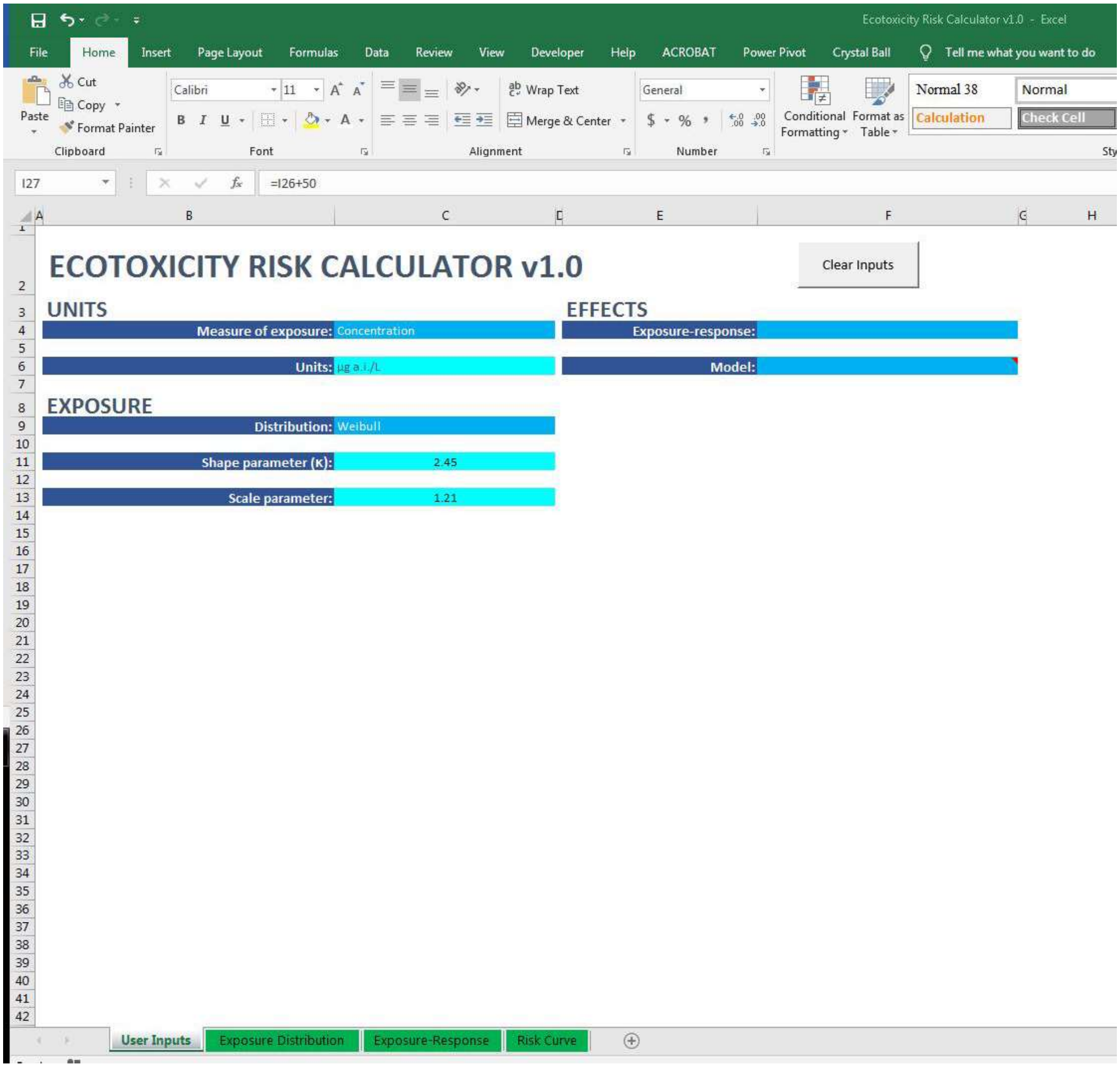

Figure 4. "User Inputs" worksheet showing, for example, the selection of a Weibull exposure distribution, with parameter inputs.

Note: Only the necessary distribution parameters can be entered for the exposure distribution. To fully clear initial inputs, press the "Clear Inputs" button on the "User Inputs" worksheet.

The entered exposure distribution can be viewed as a graph in the worksheet called "Exposure Distribution" (Figure 5).

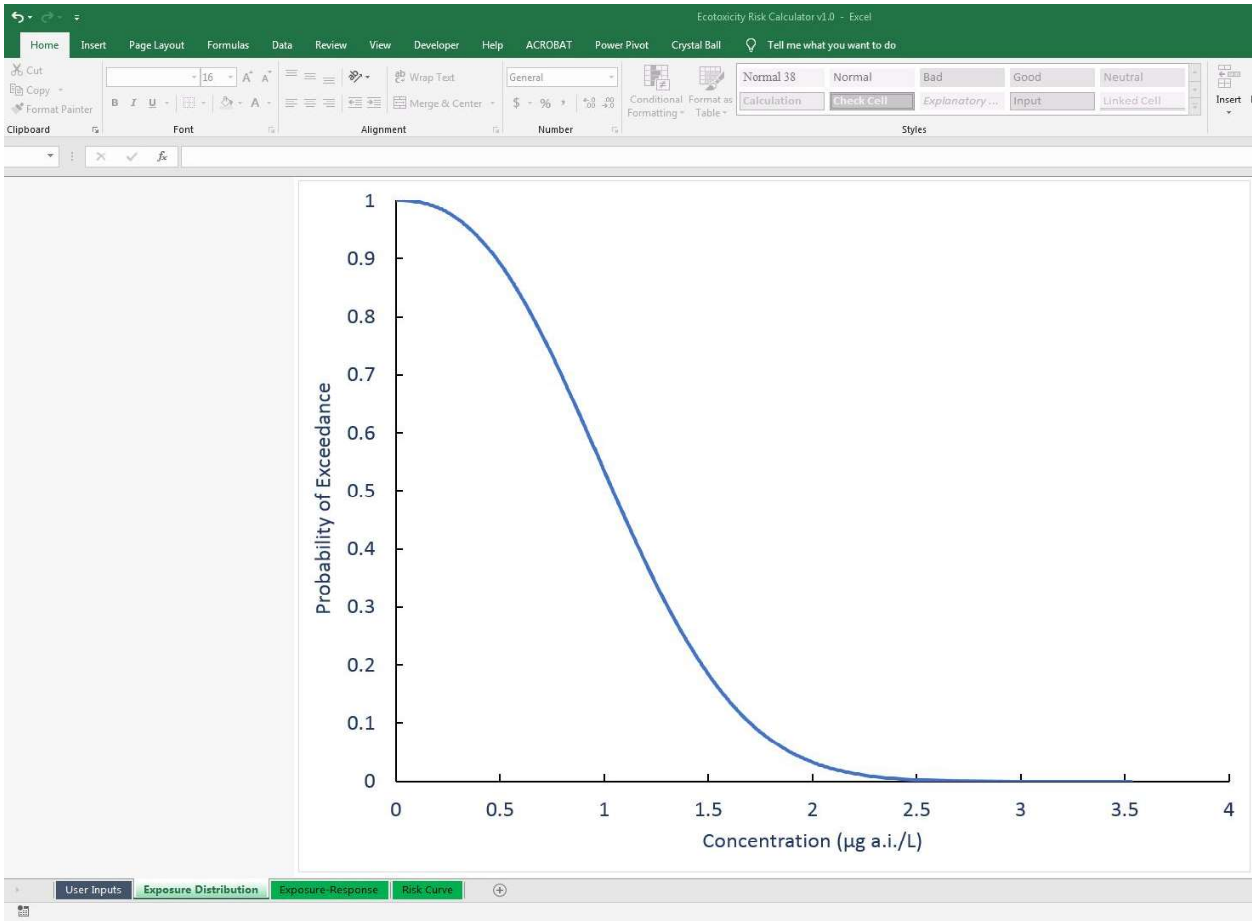

Figure 5. "Exposure Distribution" worksheet showing, for example, the Weibull distribution specified in Figure 4 as probability of exceedance versus concentration.

5.0 ENTERING EFFECTS INFORMATION

The user begins by selecting either "Single species" or "Taxon sensitivity distribution" to describe the exposure-response data (cell F4 on the "User Inputs" worksheet). "Single species" indicates that the data are from a single toxicity test conducted on individuals of one species. If "Single species" is selected the user should also select the type of measured response (i.e., survival, growth or reproduction) in cell F8 on the "User Inputs" worksheet.

The selection of "Taxon sensitivity distribution" for the exposure-response relationship indicates that the data specify a common toxicity endpoint from a number of species tests. If "Taxon sensitivity distribution" is selected, the user should also select the toxicity endpoints and taxon level (e.g., species or genus) being considered (cells F24 and F26 on the "User Inputs" worksheet).

In the Ecotoxicity Risk Calculator v1.0, the user has three options with respect to exposure-response distributions:

1) Enter an already established parametric exposure-response relationship (e.g., from a toxicity test report or statistical output from a software package).
2) Enter binomial effects data (e.g., mortality) and have a probit/normal distribution fit to the data by a maximum likelihood estimation (MLE) method.
3) Enter toxicity endpoints for a range of taxa and have a lognormal distribution fit to the data by MLE (e.g., species sensitivity distribution).

Details of each of these options are provided below.

4.1. Option 1: Parametric Exposure-Response Distributions

If the user already has a probit/normal, logistic, Gumbel, Weibull or Gompertz/extreme value exposure-response distribution that has been fit to the toxicity data, the distribution parameters can be entered directly into the Ecotoxicity Risk Calculator v1.0 (Figure 6). Details of the built-in distributions are provided in Section 7 (Computational Details). For all distributions, log10 of the measured exposure values is assumed except for the Weibull distribution. This approach prevents estimated effects at exposure values at or below zero.

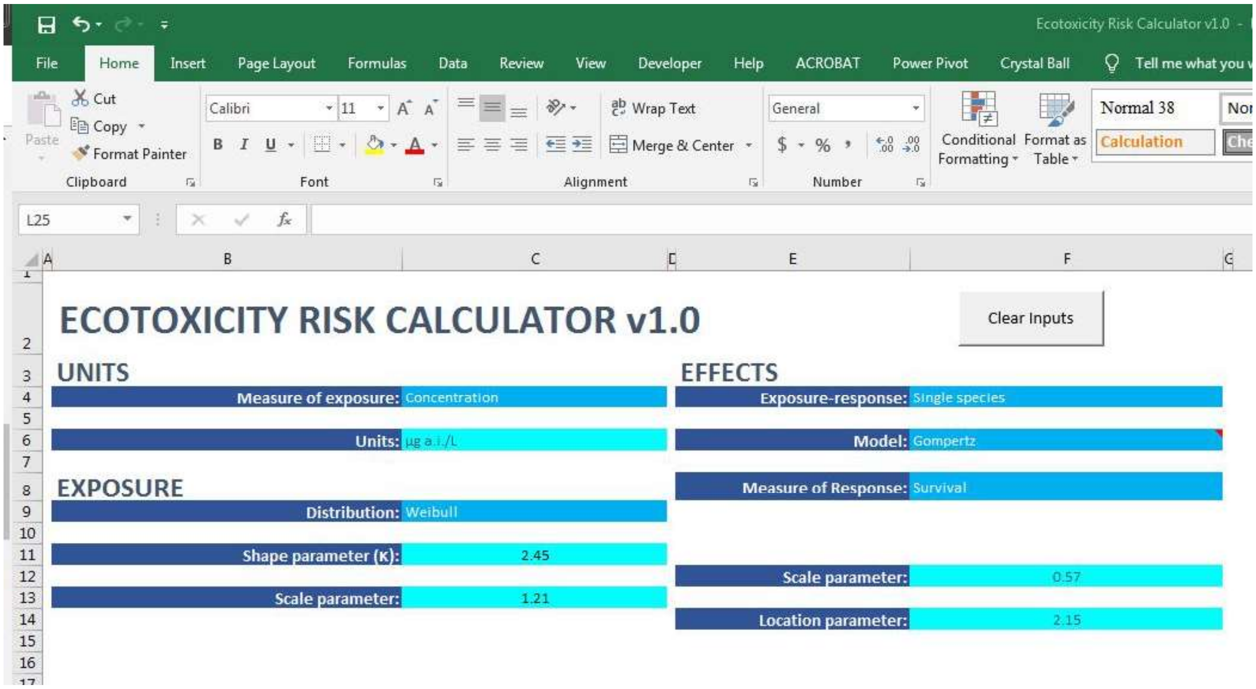

Figure 6. "User Inputs" worksheet showing, for example, the selection of a single species Gompertz concentration-response curve.

For the probit/normal distribution, three methods of describing the distribution are available (Figure 7):

1) The user can enter the parameters in terms of the tolerance distribution. That is, they can enter the location parameter (μ) and scale parameter (μ), for the normal distribution associated with the distribution fit to response vs. log10(exposure).
2) The user can enter the parameters in terms of the linear probit vs. log10(concentration), by entering the predicted median effects level (e.g., LC50) and the associated slope.
3) Finally, as is common with continuous responses, a normal response curve can also be expressed in terms of an estimated exposure level associated with a specified percent effect (e.g., ER25), and the scale parameter, σ, following Bruce and Versteeg (1992). Because the 25% effect level is commonly calculated for terrestrial plant response, this parameter estimate is included in the Ecotoxicity Risk Calculator v1.0.

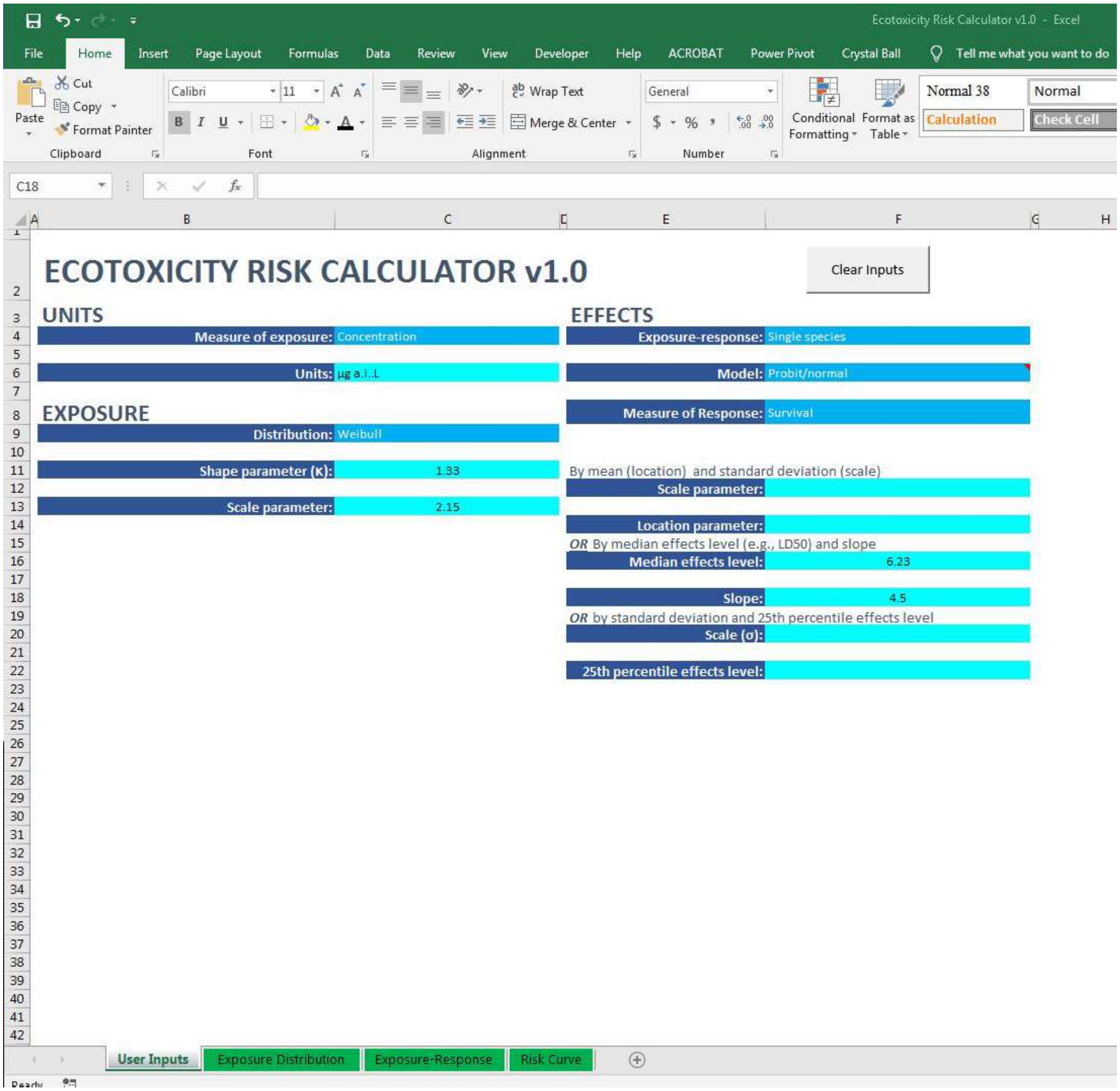

Figure 7. "User Inputs" worksheet showing, for example, the selection of a single species probit concentration-response curve input as LC50 and slope.

For all other pre-defined parametric exposure-response curves, the user enters the location and scale parameters based on log10(exposure) (Figure 6), or in the case of the Weibull distribution the scale and shape parameters based on arithmetic exposure units. Specifics of the available exposure-response distributions are found in Section 7 (Computational Details).

The input effects distribution can be found in graphical form in the "Exposure-Response" worksheet with the input distribution parameters provided in a text box on the figure (Figure 8).

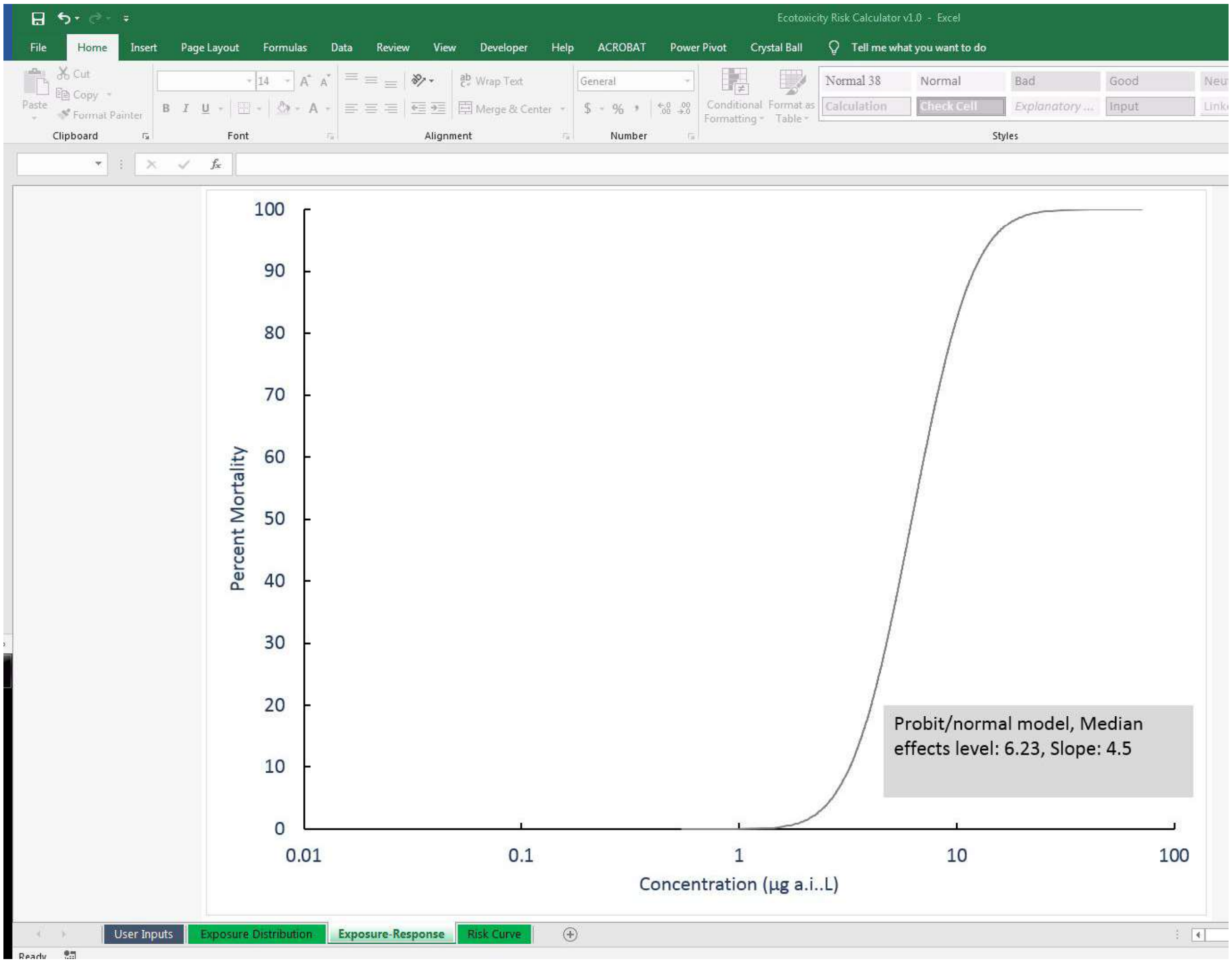

Figure 8. "Exposure-Response" worksheet showing, for example, the single species probit concentration-response curve input in Figure 7.

Note: Only one set of input parameters may be entered. To fully clear initial inputs press the "Clear Inputs" button on the "User Inputs" worksheet.

4.2. Option 2: Binomial Effects Data

To enter binomial effects data to which a probit distribution is fit by maximum likelihood estimation of parameters, "Single species" must be selected for the exposure-response (cell F4 on the "User Inputs" worksheet). The user then selects, "Estimate probit/normal distribution with data" in cell F6 of the "User Input" worksheet. The user should then select the measure of response (survival, growth or reproduction) in cell F8 on the "User Inputs" worksheet. A range of cyan cells will have appeared in columns K through M, where the user can enter binomial toxicity data (e.g., dead/alive, emerged/not emerged, birth/no birth; Figure 9).

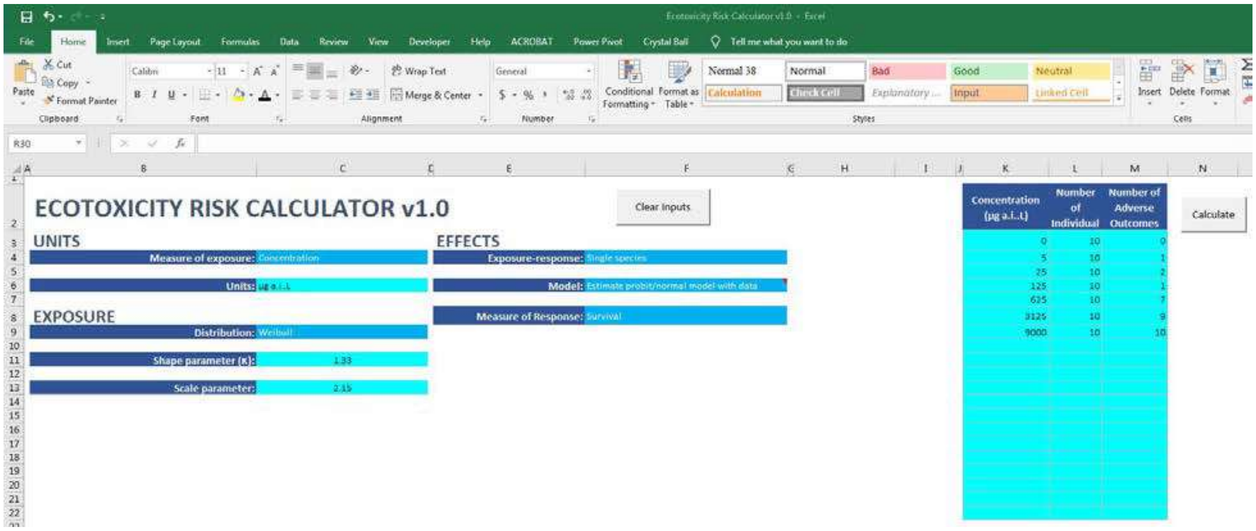

Figure 9. "User Inputs" worksheet showing the selection to "Estimate probit/normal model with data" and the range of cells that appear where data can be entered.

Note: The Ecotoxicity Risk Calculator v1.0 does not make adjustments for adverse outcomes in controls. If adjustments are required (e.g., Abbot's formula), they should be made prior to entering the data in the calculator.

Once the data have been entered, the user presses the "Calculate" button. This action invokes the Excel Solver add-in to maximize the likelihood function by changing estimates of μ and σ for the probit distribution assuming log10(exposure) as the predictor variable.

The resulting distribution can be found on the "Exposure-Response" worksheet with the fitted distribution parameters, Pearson Chi-square statistic and p-value (p-values $\geq$0.10 are generally considered acceptable; Figure 10). If the p-value is less than 0.10, we recommend that a more robust statistical application be used to fit an appropriate distribution to the data (e.g., SAS Software®). The Pearson Chi-square test calculations are provided in Section 7 (Computational Details).

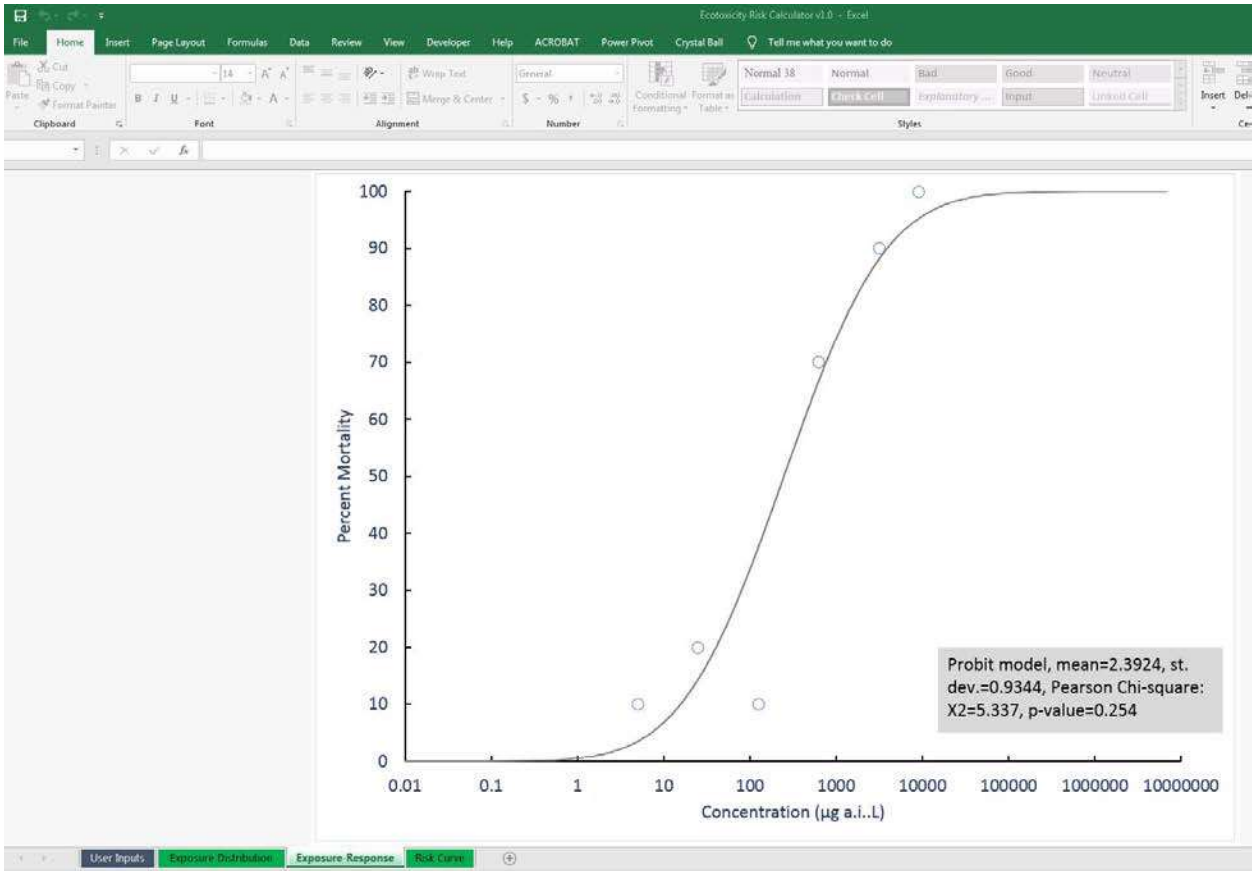

Figure 10. “Exposure-Response” worksheet showing, for example, the single species probit concentration-response curve fit to the data entered in Figure 8.

4.3. Option 3: Taxon-Specific Toxicity Endpoints

To enter toxicity endpoints to which a probit/normal distribution is fit by maximum likelihood estimation of parameters, “Taxon sensitivity distribution” must be selected for the exposure-response (cell F4 on the User Inputs spreadsheet). The user selects, “Estimate probit/normal distribution with data” in cell F6 of the “User Input” worksheet. A range of cyan cells will have appeared in columns H and I on the “User Inputs” worksheet. Here, the user can enter taxa and their associated toxicity endpoints in order from lowest to highest. Ensure that the type of endpoints and taxon level are specified in cells F24 and F26, respectively, on the “User Inputs” worksheet (Figure 11).

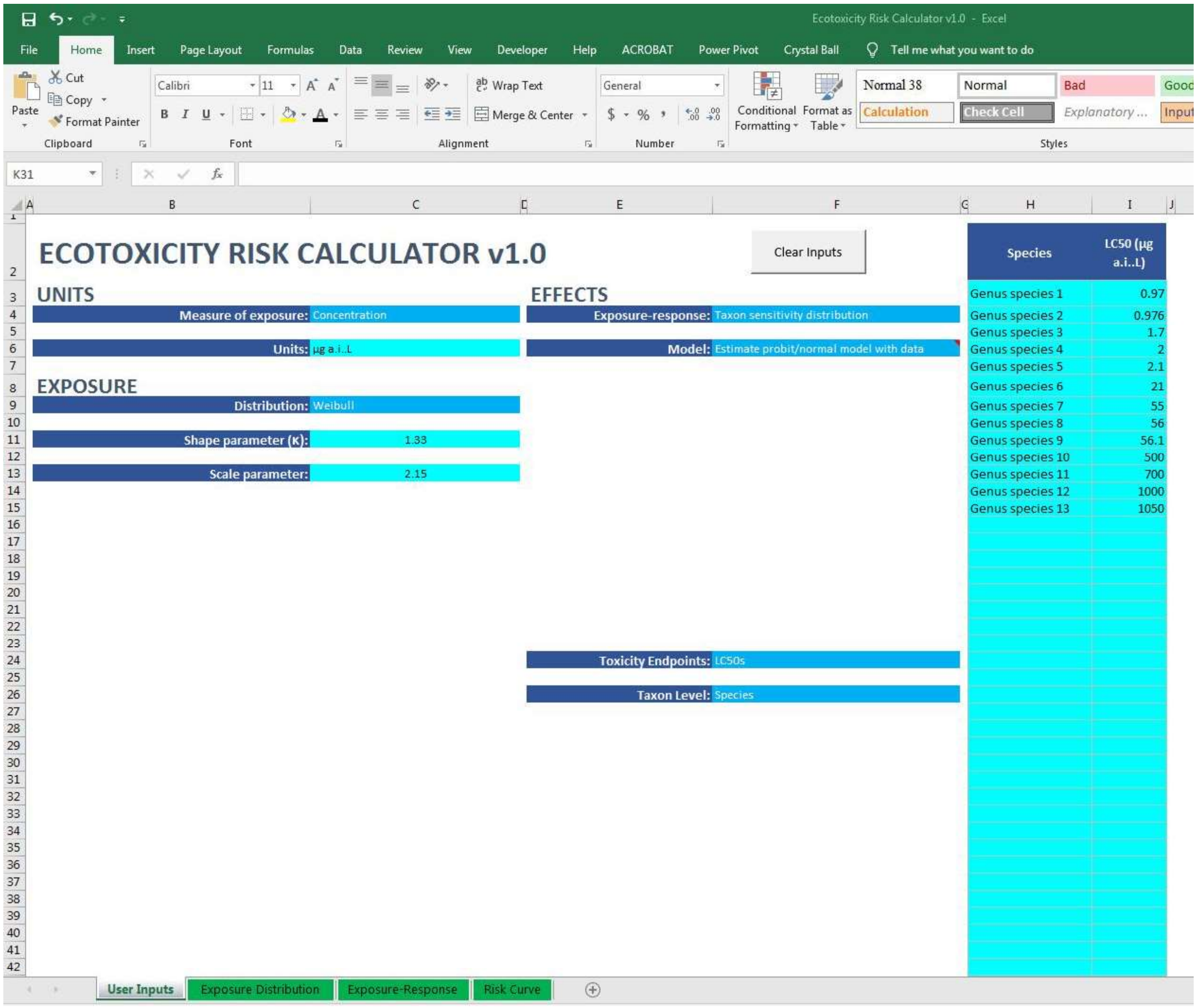

Figure 11. "User Inputs" worksheet showing how to enter data to estimate a taxon sensitivity distribution.

The sensitivity distribution is presented on the "Exposure-Response" worksheet with the taxa labels and the Anderson-Darling statistic and p-value for goodness-of-fit (Figure 12). Generally, p-values ≥0.10 are considered acceptable. If the p-value is less than 0.10, we recommend that a more robust statistical application be used to fit an appropriate distribution to the data (e.g., SAS Software®). The Anderson-Darling test calculations are provided in Section 7 (Computational Details).

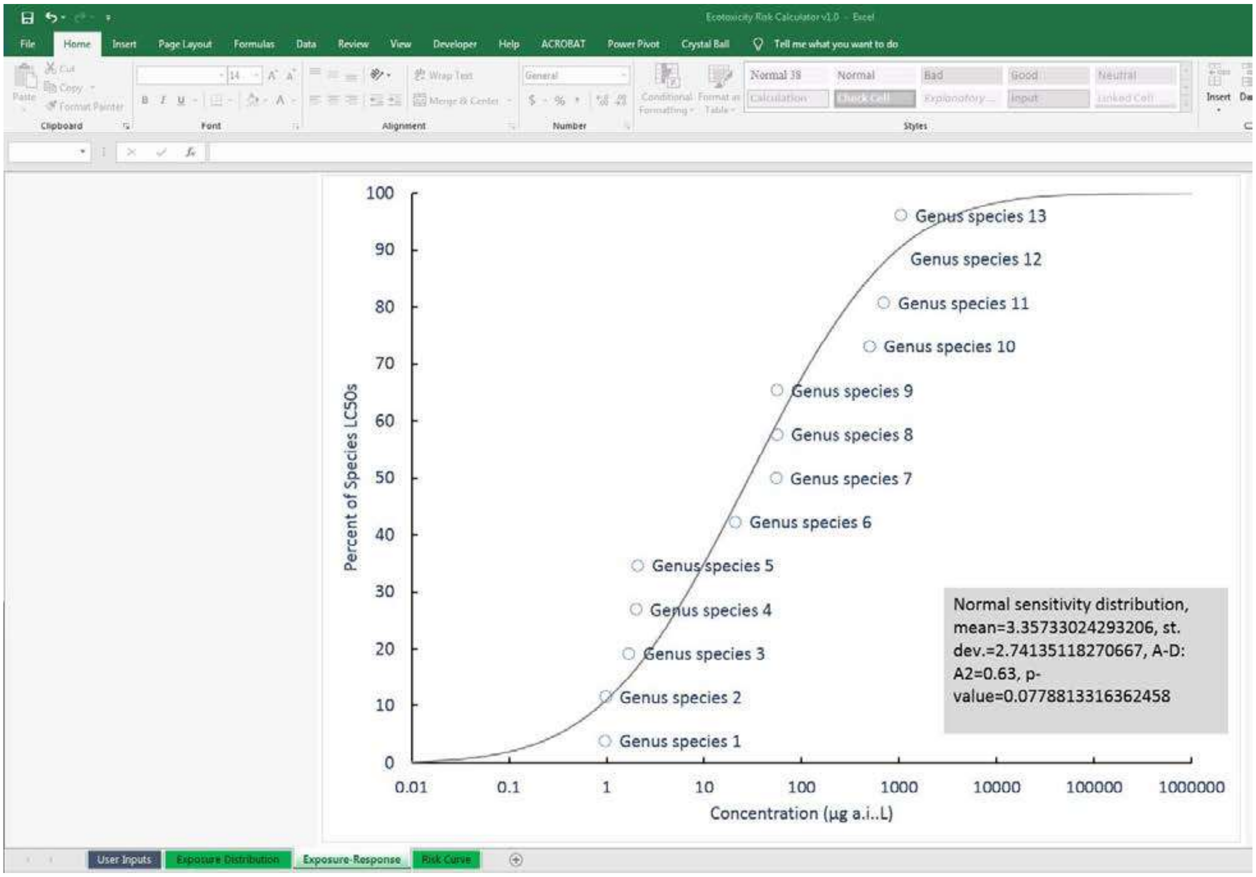

Figure 12. "Exposure-Response" worksheet showing the species sensitivity distribution fit to the data shown in Figure 11.

6.0 RISK CURVE

Once the exposure distribution and exposure-response distributions have been specified, the risk curve is automatically generated. It can be found on the "Risk Curve" worksheet in graphical form showing probability of exceedance versus magnitude of effect. Summary statistics are also provided in the "Ecotoxicity Risk Estimates" table in columns O and P of the "Risk Curve" worksheet (Figure 13). In this table, the user will find the exceedance probabilities associated with effects levels in 5% increments, as well as the calculated area under the curve (AUC) and maximum risk product. The AUC is the estimated integral of the risk curve from 0 to 100% effect. The maximum risk product is the highest product (expressed as a percent) of probability of exceedance and magnitude of effect from the risk curve. These statistics have been used in ecological risk assessments to categorize risk (e.g., Moore et al., 2009, 2010, 2014, 2015; Whitfield Aslund et al., 2016, 2017). Details of the risk curve generation, maximum risk product and AUC calculations are provided in Section 7 (Computational Details) below.

In the risk curve figure, lines of equivalent risk product are drawn to define:

- *de Minimis*-Low risk boundary (risk product=0.25%; AUC = 1.75%),
- Low-Intermediate risk boundary (risk product=2%; AUC = 9.82%), and
- Intermediate-High risk boundary (risk product=10%; AUC = 33.0%).

Categorization can be based on maximum risk product (i.e., the risk category would be assigned based on maximum risk product relative to the boundary lines). Alternatively, the risk categorization can be based on the AUC of the risk curve. If the AUC falls between the equivalent areas falling under two neighbouring boundary lines, as described in Moore et al. (2010), Moore et al. (2014) and Whitfield Aslund et al (2016), then the risk category would be the greater of these two categories.

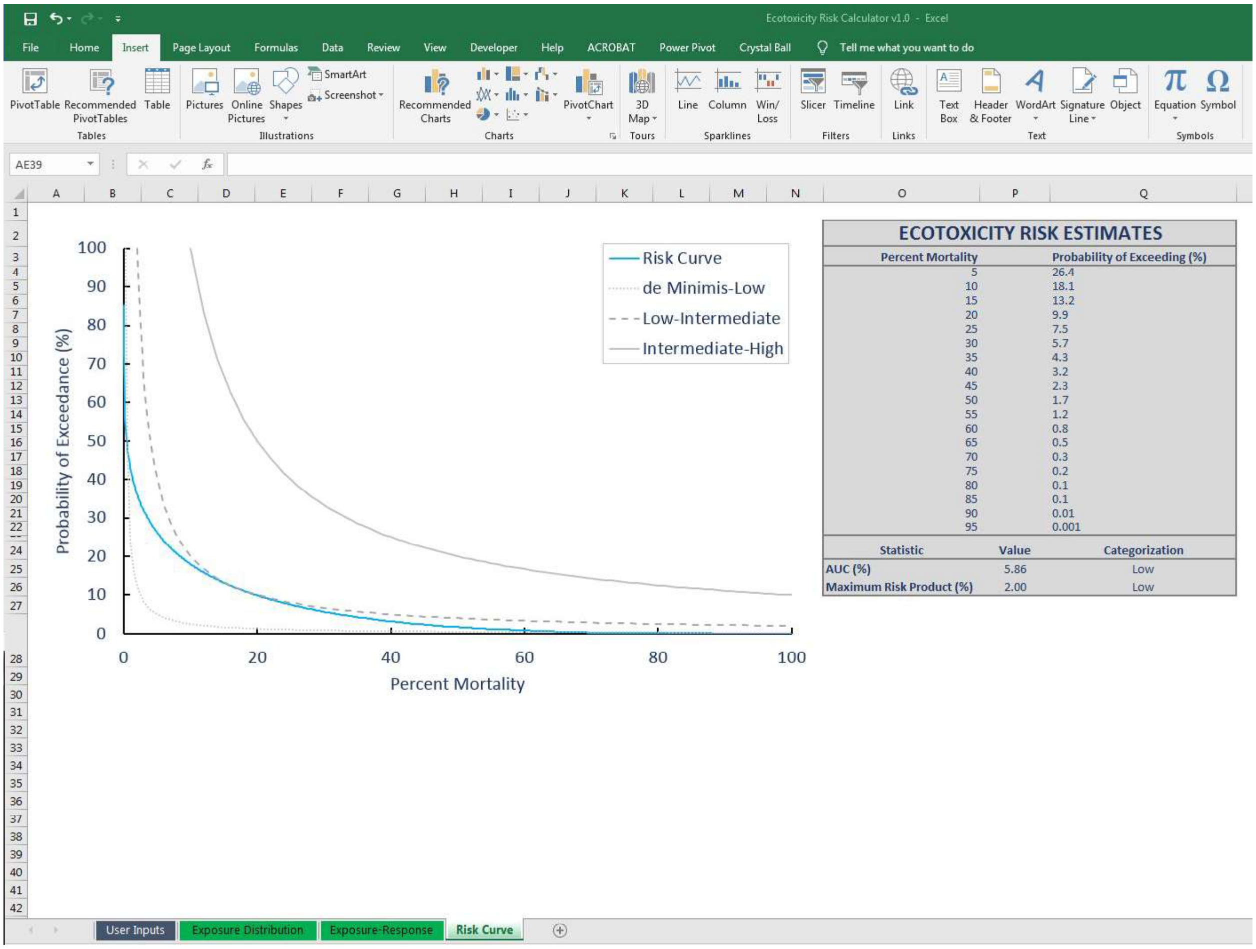

ECOTOXICITY RISK ESTIMATES

Percent Mortality	Probability of Exceeding (%)
5	26.4
10	18.1
15	13.2
20	9.9
25	7.5
30	5.7
35	4.3
40	3.2
45	2.3
50	1.7
55	1.2
60	0.8
65	0.5
70	0.3
75	0.2
80	0.1
85	0.1
90	0.01
95	0.001

Statistic	Value	Categorization
AUC (%)	5.86	Low
Maximum Risk Product (%)	2.00	Low

Figure 13. "Risk Curve" worksheet showing a risk curve generated with a survival concentration-response curve and a modeled exposure distribution.

7.0 COMPUTATIONAL DETAILS

This chapter provides details of the calculations used in the Ecotoxicity Risk Calculator v1.0 to generate risk curves.

7.1. Exposure Distributions

The user can select one of three built-in exposure distributions: normal, lognormal or Weibull. The inverse of the cumulative distribution functions, as described below are used to estimate the exposure levels associated with a full range of probabilities of exceedance on the "Exposure Distributions" spreadsheet in the Ecotoxicity Risk Calculator v1.0. These values are then used in the risk curve calculations on the "Risk Curve" worksheet.

For the normal distribution, the user enters the location and scale parameters, which we denote as μ and σ, respectively. Using these parameters, the NORM.INV(probability, mean, standard_dev) function in MS Excel® is selected. Here, probability refers to the probability of exceedance, the mean is equivalent to μ and standard_dev is equivalent to σ. This function employs the inverse of **Equation 1** (the normal cumulative distribution function) and provides the exposure level associated with the input probability of exceedance.

$$p = f(x;\mu,\sigma) = \int_{-\infty}^{x} \frac{1}{\sqrt{2\pi\sigma}} e^{-\left(\frac{(x-\mu)^2}{2\sigma^2}\right)} dx$$

Equation 1

Where,

p	=	Probability of exceedance
x	=	Exposure level in selected units
μ	=	Location parameter and mean
σ	=	Scale parameter and standard deviation

For the lognormal distribution, the user also enters the location and scale parameters, which we denote again as μ and σ, respectively. In this case, however, μ represents the mean of ln(x) and σ represents the standard deviation of ln(x). The LOGNORM.INV(probability, mean, standard_dev) function in MS Excel® is employed, which applies the inverse of Equation 1, except x is replaced with ln(x). Again, here probability refers to the probability of exceedance, mean is equivalent to μ, and standard_dev is equivalent to σ.
For the Weibull distribution, the user enters a scale parameter, λ, and a shape parameter, κ. These parameters are used to calculate the inverse of the cumulative distribution function:

$$p = f(x;\ \lambda, \kappa) = 1 - e^{-\left(\frac{x}{\lambda}\right)^{\kappa}}$$

Equation 2

Where,

p	=	Probability of exceedance
x	=	Exposure level in selected units
$\boldsymbol{\lambda}$	=	Scale parameter
$\boldsymbol{\kappa}$	=	Shape parameter

7.2. Exposure-Response Distributions Based on User Input Parameters

The user can select from five built-in exposure-response distributions. The first is the probit distribution.

$$p = f(x; \mu, \sigma) = \int_{-\infty}^{x} \frac{1}{\sqrt{2\pi\sigma}} e^{-\left(\frac{(x-\mu)^2}{2\sigma^2}\right)} dx$$

Equation 3

Where,

p	=	probability of adverse outcome
x	=	log base 10 of exposure
μ	=	location parameter
σ	=	scale parameter

The second available distribution is the **logistic distribution:**

$$p = f(x; \mu, \sigma) = \frac{1}{1 + e^{-\frac{x-\mu}{\sigma}}}$$

Equation 4

Where,

p	=	probability of adverse outcome
x	=	log base 10 of exposure
μ	=	location parameter and mean
σ	=	scale parameter

The third available distribution is the **Gompertz/extreme value distribution:**

$$p = f(x; \mu, \sigma) = 1 - e^{-e^{\frac{x-\mu}{\sigma}}}$$

Equation 5

Where,

p	=	probability of adverse outcome
x	=	log base 10 of exposure
μ	=	location parameter and mean
σ	=	scale parameter

The fourth available distribution is the **Gumbel distribution:**

$$p = f(x; \mu, \sigma\ ) = e^{-e^{\frac{(\mu-x)}{\sigma}}}$$

Equation 6

Where,

p	=	probability of adverse outcome
x	=	log base 10 of exposure
μ	=	location parameter and mean
σ	=	scale parameter

Finally, the user may select the **Weibull distribution:**

$$p = f(x) = 1 - e^{-\left(\frac{x}{\lambda}\right)^k}$$

Equation 7

Where,

p	=	Probability of exceedance
x	=	Exposure level in selected units
$\boldsymbol{\lambda}$	=	Scale parameter
$\boldsymbol{\kappa}$	=	Shape parameter

7.3. Probit Analysis for Binary Effects Data

In the Ecotoxicity Risk Calculator v1.0, the user can fit a probit distribution to binary effects data. The distribution is fit by employing the built-in Solver analysis tool in MS Excel® to maximize the log likelihood function defined as:

$$\sum_{i=1}^{k} w_i \left[r_i \ln(p_i) + (n_i - r_i)\ln(1 - p_i)\right]$$

Equation 8

Where,

k	=	number of treatments
w_i	=	weight of the ith treatment
r_i	=	number of adverse outcomes in the ith treatment
p_i	=	predicted proportion of adverse outcomes in the ith treatment
n_i	=	number of trials/individuals in the ith treatment

This is equivalent to the maximum likelihood method employed in both CETIS™ (v1.8 Tidepool 2013) and PROC PROBIT in SAS Software® (SAS 9.3, SAS/STAT 12.1) to fit probit exposure-response distributions.

The normal cumulative distribution function is employed in the probit analysis, with the following definition:

$$p = f(x; \mu, \sigma) = \int_{-\infty}^{x} \frac{1}{\sqrt{2\pi\sigma}} e^{-\left(\frac{(x-\mu)^2}{2\sigma^2}\right)} dx$$

Equation 9

Where,

p	=	probability of adverse outcome
x	=	log base 10 of exposure
μ	=	location parameter
σ	=	scale parameter

This equation is implemented using the MS Excel® function, NORM.DIST(x, mean, standard_dev, cumulative), where mean and standard_dev are equivalent to μ and σ in **Equation 9**.

Pearson Chi-square Test for Lack-of-Fit

For the probit distribution fit to the binomial data, the Pearson Chi-square statistic is calculated with **Equation 10**.

$$x_P^2 = \sum_{i=1}^{k}\sum_{j=1}^{m}\frac{\left(r_{ij} - n_i p_{ij}\right)^2}{n_i p_{ij}}$$

Equation 10

Where,

m	=	The number of possible outcomes = 2 (e.g., dead/alive)
k	=	number of treatments
r_i	=	number of adverse outcomes in the ith treatment
p_{ij}	=	predicted proportion of ith specified outcome in the jth treatment
n_i	=	number of trials/individuals in the ith treatment

The probability of a larger value for x_P^2 under the null hypothesis is calculated using the CHISQ.DIST(X, Deg_freedom, Cumulative) function in MS Excel®. Because this function returns the left-tailed probability, this value is subtracted from 1, with cumulative selected (i.e., Cumulative=TRUE). Here X is x_P^2, and Deg_freedom is set to k-2.

7.4. Taxon Sensitivity Distribution Based on Entered Toxicity Endpoints

We assume *ln(toxicity endpoint)* that values are normally distributed, and therefore the maximum likelihood estimates for the parameters of the normal distribution of ln(toxicity endpoint), are the mean and standard deviation of *ln(toxicity endpoint)* values. These calculations are expressed in **Equation 11** and **Equation 12**, respectively, and are executed in MS Excel® using the AVERAGE(Range) and the STDEV.S(Range) functions where the range consists of all the ln(toxicity endpoint) values.

$$\bar{x} = \frac{\sum_{i=1}^{t} ln(endpoint_i)}{n}$$

Equation 11

Where,

$\bar{x}$	=	The mean of *ln(endpoint)* values
t	=	The number of taxa for which there are endpoints
$endpoint_i$	=	The endpoint for the ith taxon
n	=	number of trials/individuals in the ith treatment

$$s = \sqrt{\frac{\sum_{i=1}^{t}(ln(endpoint_i) - \bar{x})^2}{n-1}}$$

Equation 12

Where,

s	=	Sample of *ln(toxicity endpoint)* values standard deviation
$\bar{x}$	=	The mean of *ln(endpoint)* values
t	=	The number of taxa for which there are endpoints
$endpoint_i$	=	The endpoint for the ith taxon
n	=	number of trials/individuals in the ith treatment

Anderson-Darling Goodness-of-Fit Test

The fit of the resulting distribution is evaluated with the Anderson-Darling statistic shown in **Equation 13**.

$$A^2 = -t - \frac{1}{t}\sum_{i=1}^{t}[(2i-1)ln(U_i) + (2t+1-2i)ln(1-U_i)]$$

Equation 13

Where,

A^2	=	The Anderson-Darling statistic
t	=	The number of taxa for which there are endpoints
U_i	=	The predicted proportion of *ln(*$endpoint$) values falling below *ln(*$endpoint_i$)

U_i is calculated using the NORM.DIST(X, Mean, St_dev, Cumulative) function in MS Excel®. X is specified as the ith *ln*($endpoint$), Mean is the value specified in **Equation 12**, *St_dev* is the value specified in **Equation 13**, and Cumulative=TRUE. The p-value is estimated for the Anderson-Darling goodness-of-fit test by first calculating an adjusted A^2 value, A*. A* is calculated as specified in **Equation 14** based on D'Agostino and Stephens (1986).

$$A^* = A^2\left(1 + \frac{0.75}{t} + \frac{2.25}{t^2}\right)$$

Equation 14

Where,

A^2	=	The Anderson-Darling statistic
A^*	=	The adjusted Anderson-Darling statistic
t	=	The number of taxa for which there are endpoints

A* can be looked up in Table 4.9 in D'Agostino and Stephens (1986) to determine the most appropriate equation for estimating p and q, the probability of a more extreme value under the

null hypothesis, and the probability of a less extreme value under the null hypothesis (q=1-p), respectively. These calculations are summarized in **Equations 15** through **18** below.

If A*<0.2, we calculate:

$$ln(q) = -13.436 + (101.14A^*) - (223.73A^{*2})$$

Equation 15

If 0.2<A*<0.34, we calculate:

$$ln(q) = -8.138 + (42.796A^*) - (59.938A^{*2})$$

Equation 16

If 0.34< A*<0.6, we calculate:

$$ln(p) = 0.9177 - (4.279A^*) - (1.38A^{*2})$$

Equation 17

Finally, if A*>0.6, we calculate:

$$ln(p) = 1.2937 - (5.709A^*) + (0.0186A^{*2})$$

Equation 18

7.5. Risk Curve Calculations

The risk curve is generated by first dividing the exposure-response distribution into increments of 0.1% effect (with finer increments in the tails; e.g., 0.0001, 0.001 and 0.01% in the lower tail). The estimated probability of exceeding the specified exposure level is determined using VLOOKUP(Lookup_value, Table_array, Col_index_num). Here, the Lookup_value is the exposure value associated with the specified effects level, the Table_array is a table containing exposure values with the probability of exceedance, where probability of exceedance is found in the specified column (Col_index_num). Because the exact exposure value will not necessarily be in the table, VLOOKUP finds an approximate match and will select the exposure value closest to but not exceeding the specified value. Estimates are negligibly different from exact matches and err on the conservative side by providing only slightly higher probabilities of exceedance than an exact match. Incrementally, risk product is calculated by multiplying the level of effect by the probability of exceedance (expressed as a percentage). The total area under the curve (AUC) is calculating using the trapezoidal rule (**Equation 19**) to approximate the area in each 0.1% increment of the exposure-response curve.

$$\int_{0}^{100} f(x)dx \approx \sum_{i=1}^{N} \frac{f(x_{i-1}) + f(x_i)}{2} \Delta_{x_i}$$

Equation 19

Where,

$f(x)$	=	The probability or exceeding effects level x
N	=	The number of increments being considered = 1,004
$f(x_{i-1})$	=	The probability of exceeding the previous effect level
$f(x_i)$	=	The probability of exceeding the current effect level
Δ_{x_i}	=	The change in effects level of the increment

8.0 REFERENCES

Brain, R.A., R.S. Teed, J. Bang, P. Thorbek, J. Perine, N. Peranginangin, M. Kim, T. Valenti, W. Chen, R.L. Breton, S. Rodney and D.R.J. Moore. 2015. Risk assessment considerations with regard to the potential impacts of pesticides on endangered species. Integrated Environmental Assessment and Management 11(1): 102-117.

Bruce, R.D. and D.J. Versteeg. 1992. A statistical procedure for modeling continuous toxicity data. Environmental Toxicology and Chemistry 11: 1485-1494.

D.Agostino. R.B. and M.A. Stephens. 1986. Goodness-of-Fit Techniques. CRC Press, Taylor & Francis Group, Boca Raton, FL. 560 pp.

Moore, D.R.J., R.S. Teed, S.I. Rodney, R.P. Thompson and D.L. Fischer. 2009. Refined avian risk assessment for aldicarb in the United States. Integrated Environmental Assessment and Management 6(1): 83-101.

Moore, D.R.J., D.L. Fischer, R.S. Teed and S. Rodney. 2010. Probabilistic risk assessment distribution for birds exposed to granular pesticides. Integrated Environmental Assessment and Management 6(2): 260-272.

Moore, D.R.J., R.L. Breton, T.R. DeLong, S. Ferson, J.P. Lortie, D.B. MacDonald, R. McGrath, A.J Pawlisz, S.C. Svirsky, R.S. Teed, R.P. Thompson, T. Tucker and M. Whitfield-Aslund. 2015. Ecological risk assessment for mink and short-tailed shrew exposed to PCBs, dioxins and furans in the Housatonic River area. Integrated Environmental Assessment and Management 12(1):174-184.

Moore, D.R.J., R.S. Teed, C.D. Greer, K. Solomon and J.P. Giesy. 2014. Refined avian risk assessment for chlorpyrifos in the United States. Reviews of Environmental Contamination and Toxicology: 231: 163-217.

NRC (National Research Council). 2013. Assessing Risks to Endangered and Threatened Species from Pesticides. Committee on Ecological Risk Assessment under FIFRA and ESA. Board on Environmental Studies and Toxicology. Division on Earth and Life Studies. National Academy of Sciences, The National Academies Press, Washington, D.C.

Tidepool. 2013. CETIS (Comprehensive Environmental Toxicity Information System) User's Guide. Tidepool Scientific Software, McKinleyville, CA.

Whitfield-Aslund, M., R.L. Breton, L. Padilla, M. Winchell, K.L. Wooding, D.R.J. Moore, R.S. Teed, R. Reiss and P. Whatling. 2016. Ecological risk assessment for Pacific salmon exposed to dimethoate in California. Environmental Toxicology and Chemistry 36(2): 532-543.

Whitfield-Aslund, M., M. Winchell, L. Bowers, S. McGee, J. Tang, L. Padilla, C. Greer, L. Knopper and D.R.J. Moore. 2017. Ecological risk assessment for aquatic invertebrate communities exposed to imidacloprid as a result of labeled agricultural and non-agricultural uses in the United States. Environmental Toxicology and Chemistry 36(5): 1375-1388.
